# Integrative pigGTEx resource with GWAS reveals genetic mechanism underlying semen quality in boars

**DOI:** 10.1186/s40104-025-01237-2

**Published:** 2025-07-21

**Authors:** Xuehua Li, Qing Lin, Zhanwei Zhuang, Kai Rao, Zhili Li, Xiuguo Shang, Panjie Xia, Lin Zhu, Zhe Zhang, Yunxiang Zhao

**Affiliations:** 1 College of Animal Science and Technology, Guangxi University, Nanning, 530004 China; 2 State Key Laboratory of Swine and Poultry Breeding Industry, Guangdong Provincial Key Lab of Agro-Animal Genomics and Molecular Breeding, College of Animal Science, South China Agricultural University, Guangzhou, 510642 China; 3 College of Animal Science and Technology, Foshan University, Foshan, 528000 China; 4 Guangxi Yangxiang Co., Ltd., Guigang, 537100 China

**Keywords:** Boar, Colocalization, GWAS, Semen quality, TWAS

## Abstract

**Background:**

Semen quality is one of the most important indicators of boar reproductive performance. In the past, boar breeding has mostly emphasized characteristics such as lean meat percentage, feed conversion efficiency, and growth rate, while overlooking the genetic improvement of reproductive traits. This study employs advanced multi-omics approaches, such as transcriptome-wide association studies (TWAS) and colocalization between genome-wide association studies (GWAS) and expression quantitative trait loci (eQTLs), to provide a comprehensive understanding of the genetic mechanisms governing semen quality traits in boars.

**Results:**

Here, we collected 190,000 ejaculate records across 11 semen quality traits from 3,604 Duroc boars. The heritability of semen quality traits ranged from 0.095 to 0.343. Genetic correlations between semen quality traits varied from −0.802 to 0.661, and phenotypic correlations ranged from −0.833 to 0.776. Single-trait GWAS identified 19 independent variants, corresponding to 13 quantitative trait loci (QTLs). By integrating PigGTEx and FAANG resources, we combined TWAS and colocalization analyses to reveal genetic regulation of semen quality traits. Notably, both GWAS and colocalization analyses pinpointed the *DCAF12* as a crucial gene associated with multiple semen quality traits. Additionally, the *ZSCAN9* gene and the variant rs322211455 were found to significantly affect sperm motility (SPMOT), possibly through hypothalamic-pituitary-gonadal axis. PheWAS further highlighted an association between rs322211455 and sperm abnormality rate, demonstrating the crucial role of *ZSCAN9* in male fertility.

**Conclusion:**

This study reveals the genetic basis and regulatory mechanisms underlying semen quality traits in Duroc boars, identifying key candidate genes such as *DCAF12* and *ZSCAN9*. These findings provide important insight into the genetic regulation of semen quality in boars.

**Supplementary Information:**

The online version contains supplementary material available at 10.1186/s40104-025-01237-2.

## Introduction

Boar is a biomedical model of male infertility in humans [1, 2]. The reproductive systems, semen composition, and physiological mechanisms share similarities between boar and human, making boar an ideal species for investigating the genetic regulation of male infertility [3]. Additionally, boar is also a critical genetic resource in hybrid breeding programs, impacting the growth and reproductive performance of offspring [4]. Conventional breeding strategies have primarily focused on traits such as growth rate, backfat thickness, and feed conversion efficiency in Duroc boars, while relatively neglecting the selection for reproductive performance. Semen quality is one of the most important reproductive indices corresponding to male fertility. The advancement of artificial insemination (AI) technologies has facilitated the widespread collection of semen phenotypes, enabling detailed investigations into the genetic architecture of semen quality traits [5–7]. Consequently, deciphering the genetic basis of semen quality is essential for addressing human male infertility but also enhancing reproductive efficiency in farm animals.

GWAS have identified tens of thousands of genetic variations associated with semen quality traits in humans [8–10], cattle [11–13], poultry [14, 15], and pigs [16–18]. Several QTLs for semen quality traits are also listed in the PigQTL Database (https://www.animalgenome.org/cgi-bin/QTLdb/index) [19]. However, these QTLs primarily focus on common semen traits such as semen volume, concentration, and motility, while more complex sperm morphological features remain underexplored in genetic studies. Furthermore, most of the identified variants from GWAS are located in non-coding regions [20], and the genetic mechanisms underlying semen quality are not fully elucidated due to complexities like genomic linkage disequilibrium (LD), limited sample sizes, and the lack of functional genomic data [21, 22]. These complexities of genetic architecture often impede the elucidation of regulatory mechanisms on complex traits. With the continuous development of functional genomics resources for farm animals, such as the FAANG (Functional Annotation of Animal Genomes) project [23] and the FarmGTEx resource [24], valuable regulatory annotations are now available to explore the genetic mechanisms of complex traits. Integrative analysis approaches, including TWAS and colocalization analysis, have gained widespread adoption [25, 26]. Despite these advancements, livestock GWAS studies are often constrained by limited sample sizes and the narrow scope of traditional phenotypes, typically involving only a few hundred individuals and traditional semen traits, due to challenges in data collection equipment and farm scale.

Here, we collected and analyzed 11 semen phenotypes and genotypes from 3,604 Duroc pigs to comprehensively identify the genetic variation of semen quality traits in pigs. After imputing genotypes to the whole genome sequence level using Pig Genomics Reference Panel (PGRP) [24], GWAS analysis was conducted to examine the genetic basis of semen quality traits. Subsequently, multi-tissue molQTL from the PigGTEx project were integrated, combined with TWAS, and colocalization analyses were conducted to systematically investigate the functional molecular mechanisms of semen quality traits in pigs.

## Materials and methods

### Animals and phenotypes

Between September 2022 and January 2024, a total of 197,159 semen records were collected from 3,604 Duroc boars of which 96.22% had complete three-generation pedigree information, covering 7,516 individuals in total across the three generations. All boars were managed by Guangxi Yangxiang Co., Ltd. (Guigang, China). The histogram of trait distribution is shown in Fig. S1, and the histogram of the number of ejaculation records per sire is shown in Fig. S2. For a more detailed description of the rearing conditions of boars, please refer to our recently published paper [27]. Semen phenotypes were measured using the UltiMate™ CASA system (Hamilton Thorne Inc., Beverly, MA, USA). This system used high-resolution phase-contrast microscopy combined with image processing algorithms to distinguish sperm morphology. For visual interpretation, the system assigns blue color to the sperm heads and red color to the necks and tails, facilitating clear morphological assessment. Eleven semen quality traits were analyzed, defined, and categorized according to the CASA system as follows:

- Semen volume (SEMVOL): The weight of each ejaculation was converted to volume using a 1:1 ratio (i.e., 1 g = 1 mL). Sperm concentration (SPCON): The number of sperm per milliliter of semen, expressed in units of 10^6^ sperm/mL. Sperm motility (SPMOT): The total number of motile sperm per unit volume, motile sperm density = sperm density × (total number of motile sperm/total number of sperm tested). Sperm progressive motility (SPPMOT): The percentage of sperm showing forward, linear movement. Sperm normality rate (SPNR): It refers to sperm with a morphologically normal structure, characterized by an oval-shaped head without deformities or defects, and a neck and tail that are free from bends or abnormalities. Sperm tail coiling (SPTACOIL): The percentage of sperm tail bend of 180° or greater along the length. Sperm tail bending (SPTABEND): The percentage of sperm tail bending exceeds 20°/µm. Sperm proximal cytoplasmic droplets (SPPCD): A droplet of cytoplasm is attached to the base of the head, where the midpiece emerges. Sperm distal cytoplasmic droplets (SPDCD): A cytoplasmic droplet attached further down the midpiece/tail from the base of the head. If the droplet is located at more than 4 µm from the base of the head, the droplet is defined as distal. Sperm distal midpiece reflex (SPDMR): The tail is wrapped around a distal cytoplasmic droplet, typically at the end of the midpiece (close to 4 µm from the end base of the sperm head), and the tail returns to the sperm head, usually becoming visible at the top of the head. Sperm functional counts (SPFCOUNT): SPFCOUNT = SEMVOL × SPCON × SPMOT × SPNR.

To ensure the reliability of the raw phenotypic data, we implemented a rigorous quality control protocol for the initial semen collection records. The exclusion criteria were as follows: SEMVOL values below 50 mL or above 650 mL, SPCON values below 10 million/mL or above 1,000 million/mL, semen collection intervals outside the range of 3 to 20 d, SPPCD values above 41.7%, and SPDCD values above 50.4% were deemed invalid and excluded from further analysis. The remaining phenotypes were filtered to remove outliers based on the 3-sigma rule.

### Genotypes

Total DNA was extracted from fresh semen samples from all 3,604 boars using a genome extraction kit (Wuhan NanoMagBio Technology Co., Ltd., China). Genotyping was performed using the Pig 80 K Functional Variants Genotyping Array (Yingzi Gene, Wuhan, China), which comprises 187,255 single-nucleotide polymorphisms (SNPs). Low-quality genotypes were filtered using PLINK v1.9 [28] with the following parameters: ‐‐mind 0.1 ‐‐geno 0.1 ‐‐maf 0.01. Genotypes were subsequently imputed from 80 K to whole-genome sequence resolution using Beagle v5.1 [29], leveraging the PGRP [24], which includes 1,602 individuals and 42,523,218 SNPs. Additional filtering was applied to remove low-quality SNPs based on criteria such as multi-allelic status, dosage R-squared < 0.8, and minor allele frequency (MAF) < 0.01. After stringent quality control, a total of 10,767,265 high-quality SNPs were retained for downstream analyses.

Then, we employed two strategies to evaluate the accuracy of genotype imputation: (1) concordance rate (CR), indicating the consistency between imputed and observed genotypes, and (2) squared correlation (R^2^), quantifying the squared correlation between the observed and imputed minor allele doses in target panels. We employed 20-fold cross-validation on the target panels among autosomes. During each set of cross-validation, we equally masked 5% SNPs from the target panels as a validation set and employed Beagle v5.1 to impute them using PGRP.

For the concordance rate between imputed and observed genotypes, the formula was as follows:

$$CR=\frac{{Genotype}_{imp}}{{Genotype}_{obs}}$$

Where ${Genotype}_{imp}$ indicates the number of imputed genotypes consistent with the observed genotypes, ${Genotype}_{obs}$ represents the total number of observed genotypes.

For the squared correlation between the observed and imputed minor allele doses, the formula was as follows:

$$Imputed\;dosage=0\times P\left(AA\right)+1\times P\left(AB\right)+2\times P\left(BB\right)$$

$$R^2={Cor}^2\left(Imputed\;dosage,Observed\;dosage\right)$$

### Genetic parameter estimation

The DMUAI module of DMU [30] software was used for genetic parameter estimation and variance component calculation of semen quality traits. Variance components and their standard errors (SEs) were estimated using the average information restricted maximum likelihood (AI-REML) algorithm. We first calculated the variance component based on pedigree information using a single-trait repeatability model. The statistical model is:

$${\varvec{Y}}={\varvec{X}}{\varvec{b}}+{\varvec{Z}}{\varvec{u}}+{\varvec{P}}{\varvec{p}}+{\varvec{e}}$$

$\boldsymbol Y$ is the vector of phenotypic values for the traits; $\boldsymbol b$ is the vector of fixed effects (e.g., year-season of ejaculation, age of pigs (days) or collection interval (days); $\boldsymbol u\sim N(0,\boldsymbol A\sigma_a^2)$ is the vector of individual additive genetic effects, with $\boldsymbol A$ and ${\sigma }_{a}^{2}$ denoting the pedigree-based additive genetic relationship matrix and additive genetic variance; $\boldsymbol p\sim N(0,\boldsymbol I\sigma_{pe}^2)$ is the vector of permanent environmental effects with ${\sigma }_{pe}^{2}$ denoting the permanent environment variance; and $\boldsymbol e\sim N(0,\boldsymbol I\sigma_e^2)$ is the vector of random residual effects, with $\boldsymbol I$ and ${\sigma }_{e}^{2}$ denoting the identity matrix and the residual variance. $\boldsymbol X$, $\boldsymbol Z$, and $\boldsymbol P$ are the incidence matrices for $\boldsymbol b$, $\boldsymbol u$, and $\boldsymbol p$, respectively.

We further estimated the genetic correlation for all semen quality trait pairs using two-trait repeatability model. The model was as follow:

$$\left[\begin{array}{c}{{\varvec{y}}}_{1}\\ {{\varvec{y}}}_{2}\end{array}\right]=\left[\begin{array}{cc}\begin{array}{c}{{\varvec{X}}}_{1}\\ \varvec{0}\end{array}& \begin{array}{c}\varvec{0}\\ {{\varvec{X}}}_{2}\end{array}\end{array}\right]\left[\begin{array}{c}{{\varvec{b}}}_{1}\\ {{\varvec{b}}}_{2}\end{array}\right]+\left[\begin{array}{cc}\begin{array}{c}{{\varvec{Z}}}_{1}\\ \varvec{0}\end{array}& \begin{array}{c}\varvec{0}\\ {{\varvec{Z}}}_{2}\end{array}\end{array}\right]\left[\begin{array}{c}{{\varvec{a}}}_{1}\\ {{\varvec{a}}}_{2}\end{array}\right]+\left[\begin{array}{cc}\begin{array}{c}{{\varvec{W}}}_{1}\\ \varvec{0}\end{array}& \begin{array}{c}\varvec{0}\\ {{\varvec{W}}}_{2}\end{array}\end{array}\right]\left[\begin{array}{c}{{\varvec{p}}{\varvec{e}}}_{1}\\ {{\varvec{p}}{\varvec{e}}}_{2}\end{array}\right]+\left[\begin{array}{c}{{\varvec{e}}}_{1}\\ {{\varvec{e}}}_{2}\end{array}\right]$$

Where $$\left[\begin{array}{c}{{\varvec{y}}}_{1}\\ {{\varvec{y}}}_{2}\end{array}\right]$$ is the vector of phenotypic value for semen quality traits. $$\left[\begin{array}{c}{{\varvec{b}}}_{1}\\ {{\varvec{b}}}_{2}\end{array}\right]$$ is the vector of fixed effects including the year of semen collection, season of semen collection, age of pigs (days) or collection interval (days). $$\left[\begin{array}{c}{{\varvec{a}}}_{1}\\ {{\varvec{a}}}_{2}\end{array}\right]\sim N(0, {\varvec{A}}\left[\begin{array}{cc}\begin{array}{c}{\sigma }_{a1}^{2}\\ {\sigma }_{a21}\end{array}& \begin{array}{c}{\sigma }_{a12}\\ {\sigma }_{a2}^{2}\end{array}\end{array}\right])$$ is the vector of additive genetic effect, $$\left[\begin{array}{cc}\begin{array}{c}{\sigma }_{a1}^{2}\\ {\sigma }_{a21}\end{array}& \begin{array}{c}{\sigma }_{a12}\\ {\sigma }_{a2}^{2}\end{array}\end{array}\right]$$ is the additive genetic variance–covariance matrix, and ${\varvec{A}}$ is the pedigree-based relationship matrix. $$\left[\begin{array}{c}{{\varvec{p}}{\varvec{e}}}_{1}\\ {{\varvec{p}}{\varvec{e}}}_{2}\end{array}\right]\sim N(0, {\varvec{I}}\left[\begin{array}{cc}\begin{array}{c}{\sigma }_{pe1}^{2}\\ {\sigma }_{pe21}\end{array}& \begin{array}{c}{\sigma }_{pe12}\\ {\sigma }_{pe2}^{2}\end{array}\end{array}\right])$$ is the vector of permanent environment effect, $$\left[\begin{array}{cc}\begin{array}{c}{\sigma }_{pe1}^{2}\\ {\sigma }_{pe21}\end{array}& \begin{array}{c}{\sigma }_{pe12}\\ {\sigma }_{pe2}^{2}\end{array}\end{array}\right]$$ is the permanent environment variance–covariance matrix. $$\left[\begin{array}{c}{{\varvec{e}}}_{1}\\ {{\varvec{e}}}_{2}\end{array}\right]\sim N(0, {\varvec{I}}\left[\begin{array}{cc}\begin{array}{c}{\sigma }_{e1}^{2}\\ {\sigma }_{e21}\end{array}& \begin{array}{c}{\sigma }_{e12}\\ {\sigma }_{e2}^{2}\end{array}\end{array}\right])$$ is the vector of residual effect, $$\left[\begin{array}{cc}\begin{array}{c}{\sigma }_{e1}^{2}\\ {\sigma }_{e21}\end{array}& \begin{array}{c}{\sigma }_{e12}\\ {\sigma }_{e2}^{2}\end{array}\end{array}\right]$$ is the residual variance–covariance matrix. $$\left[\begin{array}{cc}\begin{array}{c}{{\varvec{X}}}_{1}\\ 0\end{array}& \begin{array}{c}0\\ {{\varvec{X}}}_{2}\end{array}\end{array}\right]$$, $$\left[\begin{array}{cc}\begin{array}{c}{{\varvec{Z}}}_{1}\\ 0\end{array}& \begin{array}{c}0\\ {{\varvec{Z}}}_{2}\end{array}\end{array}\right]$$ and $$\left[\begin{array}{cc}\begin{array}{c}{{\varvec{W}}}_{1}\\ 0\end{array}& \begin{array}{c}0\\ {{\varvec{W}}}_{2}\end{array}\end{array}\right]$$ are the corresponding design matrices for the fixed effect $$\left[\begin{array}{c}{{\varvec{b}}}_{1}\\ {{\varvec{b}}}_{2}\end{array}\right]$$, additive genetic effect $$\left[\begin{array}{c}{{\varvec{a}}}_{1}\\ {{\varvec{a}}}_{2}\end{array}\right]$$ and permanent environment effect $$\left[\begin{array}{c}{{\varvec{p}}{\varvec{e}}}_{1}\\ {{\varvec{p}}{\varvec{e}}}_{2}\end{array}\right]$$.

### Repeatability GWAS

In this study, phenotypic data comprised multiple ejaculate records per individual collected across various time points. To account for this structure, a repeatability model implemented in the GMAT (V1.02) software [31] was used for association analysis. The analytical framework was based on the following model:

$${\varvec{y}}={\varvec{X}}{\varvec{b}}+{\varvec{x}}{\varvec{c}}+{\varvec{w}}{\varvec{\beta}}+{\varvec{Z}}{\varvec{g}}+{\varvec{W}}{\varvec{p}}{\varvec{e}}+{\varvec{e}}$$

Where ${\varvec{y}}$ is a vector of phenotypes for semen quality traits. ${\varvec{b}}$ is a vector of fixed effects, including the year of semen collection and season of semen collection. ${\varvec{c}}$ indicates the covariates, including the top five genotype principal components. ${\varvec{x}}$ indicates the regression coefficient of covariates. ${\varvec{\beta}}$ is the effect of SNP, and ${\varvec{w}}$ is a vector of SNP genotypes assigned a value of 0, 1, or 2 for AA, Aa, and aa, respectively. ${\varvec{g}}$~*N* (0,${\varvec{G}}{\sigma }_{g}^{2}$) is a vector of additive genetic effect, ${\varvec{G}}$ is the genomic relationship matrix built with the method of VanRaden, and ${\sigma }_{g}^{2}$ is the additive genetic variance. ${\varvec{p}}{\varvec{e}}$~*N* (0,${\varvec{I}}{\sigma }_{pe}^{2})$ is the vector of permanent environment effect, ${\sigma }_{pe}^{2}$ is the permanent environment variance. ${\varvec{e}}$~*N* (0,${\varvec{I}}{\sigma }_{e}^{2})$ is the vector of residual effect, ${\sigma }_{e}^{2}$ is the residual variance, and ${\varvec{I}}$ is the identity matrix. ${\varvec{X}}$, ${\varvec{Z}}$ and ${\varvec{W}}$ are the corresponding design matrices for the fixed effect ${\varvec{b}}$, additive genetic effect ${\varvec{g}}$ and permanent environment effect ${\varvec{p}}{\varvec{e}}$. Furthermore, the genome-wide significance threshold was set at 5 × 10^–8^; all SNPs that exceeded this threshold were considered significant for further analysis.

To assess the robustness and reproducibility of GWAS results, we performed bidirectional cross-validation using the external validation dataset of 2,000 Duroc boars to evaluate the replication rate ($\pi 1)$ of GWAS signals. The replication rate was calculated as $\pi 1=B/A$. Firstly, set $A$ comprises SNPs that reached significance (*P* < 0.001) in the discovery dataset and were also present in the validation dataset. Within set $A$, SNPs that showed nominal significance (*P* < 0.1) in validation dataset were defined as set $B$. For reciprocal validation, we repeated the analysis by interchanging the discovery and validation datasets.

### Conditional analysis and QTL definition

To obtain independent variants and define QTL, we employed GCTA-COJO [32] for the determination of independent variants in GWAS. The imputed genotypes were utilized as the reference panel for LD estimation and subsequent conditional analyses. Specifically, the parameters --maf 0.01, --cojo-slct, and --cojo-p 5e-8 were applied within the GCTA software [32] to identify independent variants. Subsequently, QTLs were defined as regions encompassing variants with an LD threshold greater than 0.8 relative to the independent variants, extending to the farthest positions on either side of the independent variant.

### Gene-based association analysis

To identify candidate genes associated with semen quality traits, gene-based association analysis was conducted based on the GWAS summary statistics using MAGMA [33]. Initially, integrated the genomic annotation of the pig genome (*Sus scrofa 11.1*, UTL: https://ftp.ensembl.org/pub/release-100/gtf/sus_scrofa/Sus_scrofa.Sscrofa11.1.100.gtf.gz.) with the combined genotypes using the parameters --annotate, --snp-loc, and --gene-loc to generate a gene annotation file. Subsequently, gene-level association analysis was performed using the parameters --gene-annot and --pval to compute the associations at the gene level. Finally, Bonferroni correction was applied to adjust the significance threshold, filtering the candidate genes in the gene-level association.

### Functional annotation and characterization

We conducted gene annotation within the QTL regions using the physical position map of the *Sus scrofa 11.1* genome from Ensembl (http://asia.ensembl.org/index.html, accessed July 6, 2024), identifying the genomic locations of all variants situated within these QTLs.

To further investigate the biological functions of these candidates, we performed Gene Ontology (GO) and Kyoto Encyclopedia of Genes and Genomes (KEGG) pathway enrichment analyses using the KOBAS platform [34]. Moreover, we carried out an extensive literature review through PubMed to examine potential associations between the candidate genes and semen quality traits.

### Integrative analysis with pigGETx

Exploring the regulatory mechanism among genetic loci, genes, and semen phenotypes is crucial for understanding the underlying biology. By integrating approaches such as TWAS and colocalization analysis, it becomes possible to investigate the regulatory pathways through which genetic variants influence semen quality traits, providing initial insights into the molecular mechanisms driving these phenotypic outcomes.

#### Transcriptome-wide association study (TWAS)

To detect the significant associations between genetically predicted gene expression levels and semen phenotypes, we retrieved the single‐tissue TWAS models from the PigGTEx resource [24]. Using summary statistics from 11 semen trait GWASs, we performed single-tissue TWAS with S-PrediXcan [35]. A Bonferroni correction for multiple testing was applied to determine the significance threshold.

#### Colocalization

To identify potential regulatory genes influencing phenotypes or to assess the likelihood of shared causal variants between phenotypes and regulatory genes, we performed Bayesian colocalization analysis using the “coloc” R package [35]. First, we obtained the summary statistics for expression quantitative trait loci (eQTLs) mapping from the PigGTEx resource. We then selected all SNPs within ± 1 Mb of the independent variants identified through conditional analysis as the colocalization SNP set. Genetic information from both GWAS and eQTL mapping results were extracted for these SNPs. The colocalization analysis was subsequently performed using the Bayesian approach with default settings. A posterior probability (PP4) greater than 0.8 was set as the threshold for moderate to strong colocalization evidence.

## Results

### The genetic parameters of semen quality traits

To explore the genetic parameters of semen quality traits, we first summarized the phenotypes in Table S1 and Fig. S1. In total, 3,604 boars with 163,140 to 197,159 records were utilized to estimate the genetic parameters. The coefficients of variation of semen quality traits ranged from 4.6% to 107.46%, indicating substantial variability across semen quality traits.

Then, the heritability of 11 semen quality traits ranged from 0.095 to 0.343, reflecting low to median heritability. For instance, SPFCOUNT had the highest heritability at 0.343 ± 0.015, while SPNR had the lowest at 0.095 ± 0.013. Uncommon semen quality traits, such as sperm tail coiling, tail bending, cytoplasmic droplets, and distal midpiece reflex, exhibited heritability values ranging from 0.137 to 0.206. This exploration of the genetic architecture of sperm morphological abnormalities not only enhanced the understanding of semen quality but also provided a reference for unconventional genetic parameters of semen.

**Fig. 1:**
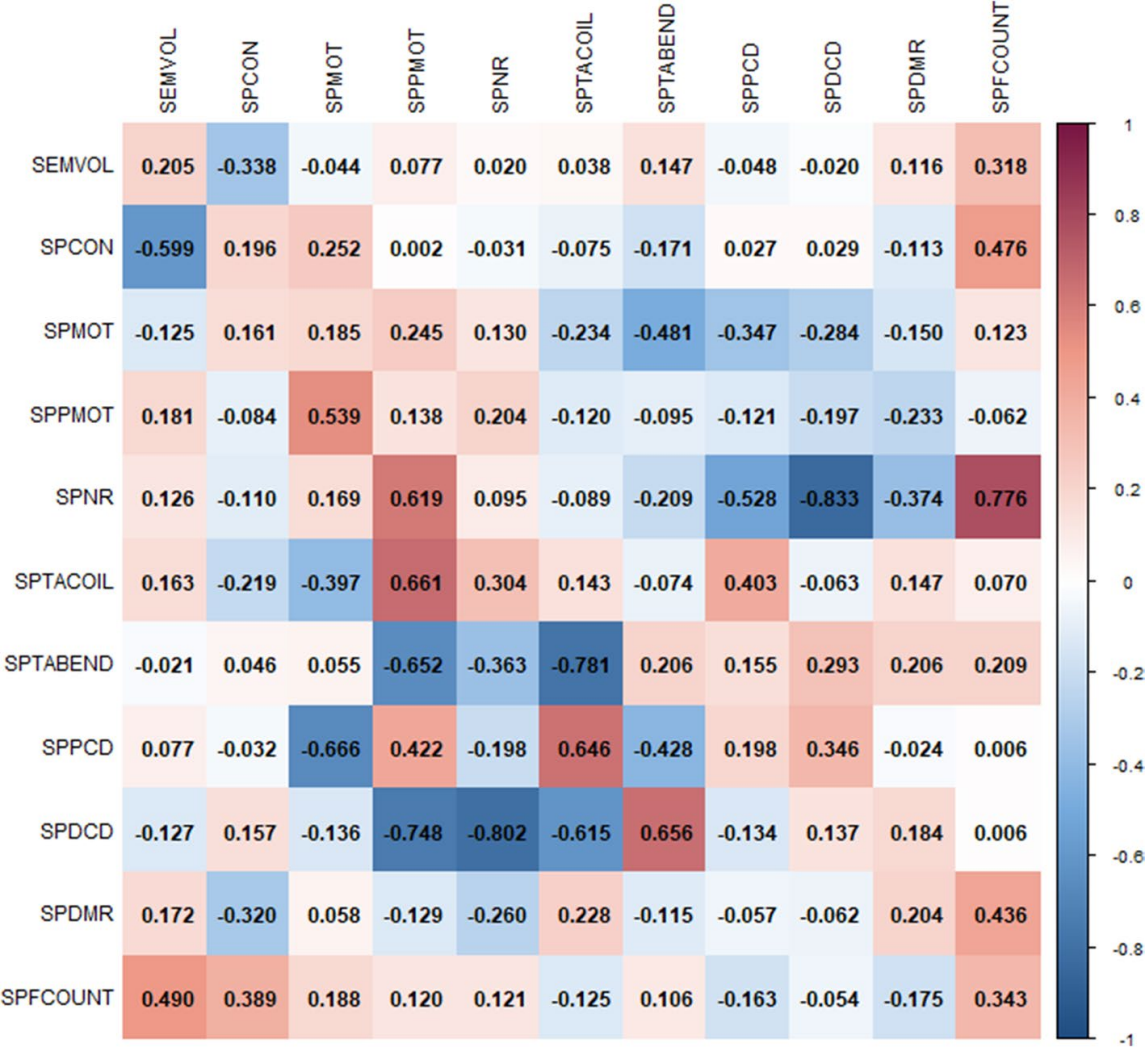
The genetic parameter estimates for semen traits are shown. The diagonal represents the heritability of each trait. The genetic correlations between traits are presented in the lower left of the diagonal, while the phenotypic correlations are shown in the upper right

Furthermore, both genetic and phenotypic correlations among 11 semen quality traits were showed in Fig. 1. The genetic correlation of semen quality traits ranged from −0.802 to 0.661, and the phenotypic correlation coefficients varied between −0.833 and 0.776, indicating a complex and interconnected genetic architecture among semen quality traits. Notably, high genetic correlations were observed among SPPMOT, SPTACOIL, and SPDCD. For instance, the genetic correlation between SPPMOT and SPTACOIL, and between SPPMOT and SPDCD were 0.661 and −0.748, respectively, while the genetic correlation between SPTACOIL and SPDCD was −0.615, indicating the potential shared genetic regulatory mechanisms.

In summary, the genetic parameters of semen quality in Duroc boars were diverse, underscoring the potential for genetic improvement. The estimation of genetic parameters for uncommon semen traits filled a critical gap in our understanding of sperm morphological abnormalities and offered valuable insights into the genetic basis of semen quality traits.

### Individual GWAS for 11 semen quality traits

To investigate the genetic architecture of semen quality traits, we carried out genome-wide association studies that were based on single-trait repeatability model. Firstly, we imputed the genotype data from the SNP array to whole-genome sequence level using Beagle v5.1 [29]. After quality control, 10,767,265 SNPs were retained for subsequent analysis. The SNP consistency and accuracy of genotype imputation were 0.994 ± 0.022 and 0.981 ± 0.085 on average, respectively, indicating the reliable imputed genotypes for GWAS analyses (Fig. S3). Kinship relationships among individuals were shown in Fig. S4. To eliminate the confounding effects of kinship on the association analysis and accurately assess the true associations between SNPs and traits, the genomic relationship matrix (GRM) was incorporated into the model. To investigate the population structure, we performed principal component analysis (PCA) based on the genotypes before and after genotype imputation (Fig. S5). The results showed that the population clustered into three groups to correct population structure of GWAS population, we added the top 5 PCAs as covariates into the GWAS model.

**Fig. 2:**
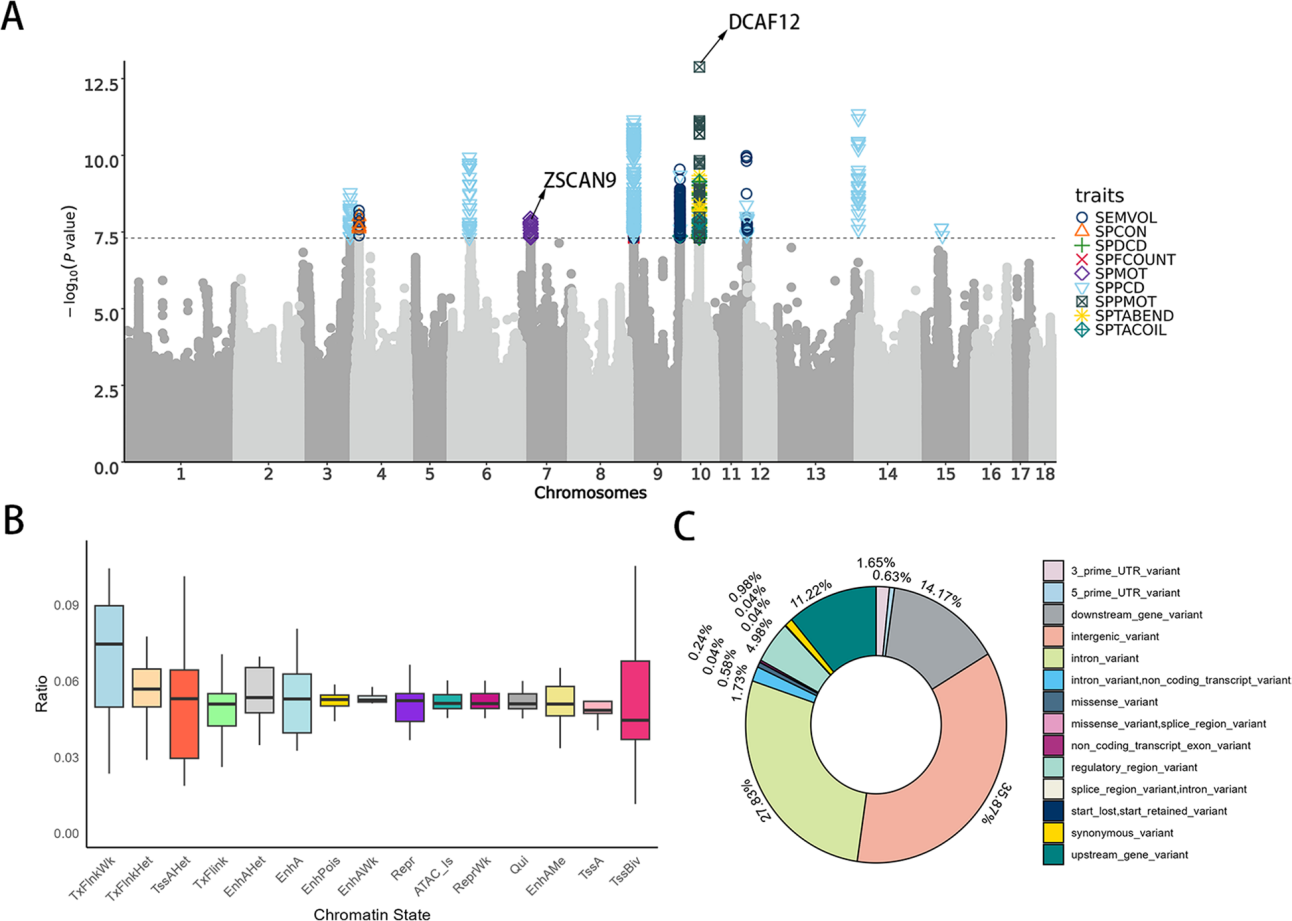
GWAS analysis identified loci associated with semen quality, along with functional annotation of the associated variants and various variant types. **A** The Manhattan plot for individual GWAS across 11 semen quality traits. The grey dashed line presents the suggested significance threshold, set at *P* = 5 × 10^–8^. The shape represents different semen quality traits. **B** The box plot for the enrichment of SNPs within the QTLs corresponding to the 11 semen quality traits from the GWAS in regulatory elements across 14 different tissues. **C** The hollow circle plot represents the enrichment of variant types within the QTLs associated with semen quality traits

Then, we conducted individual GWAS for 11 semen quality traits using GMAT (v1.02) [31] (Fig. 2A). The genomic inflator factor (λ) ranged from 0.906 to 0.974 (Table S2), indicating robust control of population stratification. In total, 1,276 significant variants were obtained across 11 GWASs. The conditional analysis using GCTA-COJO identified 19 independent variants (Table S3), corresponding to 13 QTLs. The majority of the QTLs were novel and not been listed in PigQTLdb. Although a few of these QTLs had been confirmed to be associated with traits such as litter size and teat number in previous studies, none overlap with prior studies on semen quality traits. To further assess the robustness of our GWAS result, we performed reciprocal validation using the $\pi 1$ statistic. The summary statistics about the validation population were shown in Table S4. The $\pi 1$ results were presented in Fig. S6. The $\pi 1$ value for discovery-in-validation was higher than that for validation-in-discovery, indicating the reliability of the GWAS signals in discovery population. The relatively small $\pi 1$ values may be attributed by permanent effect, environmental effects or genotype-by-environment (G×E) interactions. The high phenotypic variation in semen quality across different ejaculates from the same boar supports the notion that non-genetic factors have a strong influence.

Functional annotation of the candidate genes using KOBAS [34] revealed significant enrichment in 8 GO terms (*P *< 0.01) and 13 KEGG pathways (*P *< 0.05). Among these, the most significantly enriched pathway was protein digestion and absorption (ssc04974, *P* = 2.15 × 10^–4^). Notably, this pathway was also identified in previous studies on semen quality related to male age, where differentially expressed proteins were annotated to the same pathway, indicating a potential biological link between nutrient absorption and semen quality-related changes in reproductive function (Fig. S7 and Table S5 and S6) [36]. To explore the genomic features of variants associated with semen quality, we annotated the identified significant variants using VEP [37]. The annotation results revealed 13 variant types, with the majority (98.12%) of variants located in non-coding regions (Fig. 2C). Functional annotation revealed that SNPs were principally enriched in transcription start site (TSS) proximal transcribed regions (Fig. 2B), suggesting that the regulatory mechanisms underlying semen quality are complicated.

### Colocalization reveals *DCAF12* as a key regulator gene of male fertility in boars

**Fig. 3:**
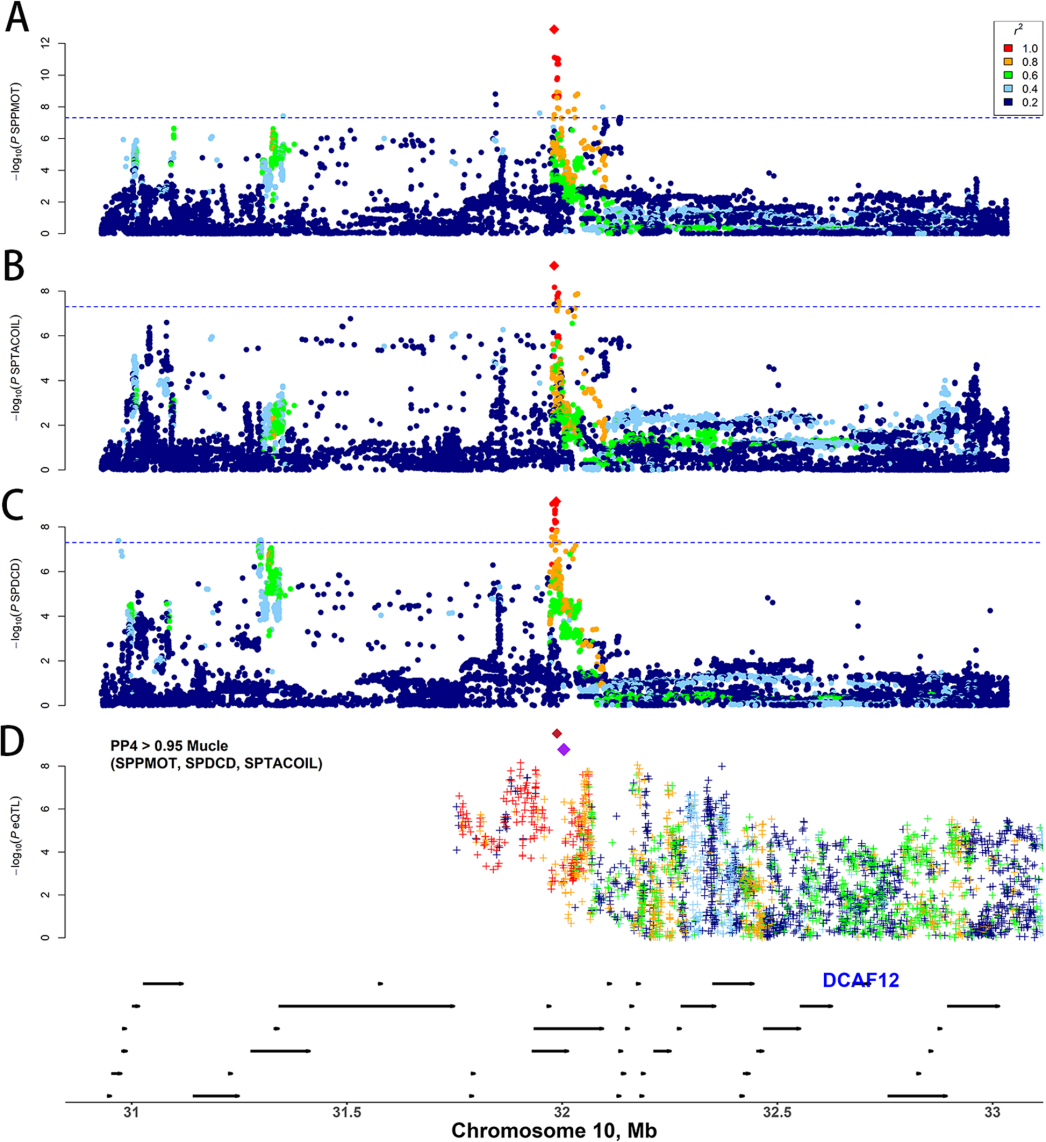
The regional Manhattan plot for the independent variant rs319824241 and eQTL mapping for *DCAF12* in muscle with colocalization. **A**–**C** The Manhattan plot for semen quality traits (SPPMOT, SPTACOIL, and SPDCD) within the QTL region on chromosome 10. The red diamond shaped point represents the independent variant rs319824241. The colors correspond to LD (*r*^2^) between the independent variant and other variants. **D** The eQTL mapping results for *DCAF12* in muscle tissue. Red diamond markers represent the lead eQTL for *DCAF12* in muscle, while purple diamond markers represent the lead SNPs within the QTL region. The colors correspond to LD (*r*^2^) between the independent variant and other variants. The bottom panel displays the gene positioning, with arrows indicating the direction of gene expression

Individual GWAS identified a 32.23 kb (31,907,024–31,939,255) QTL on chromosome 10 that simultaneously influences three semen quality traits: SPPMOT, SPTACOIL, and SPDCD (Fig. 3A–C). Annotation of the SNPs within this QTL revealed that all variants were located in non-coding regions, with the majority positioned in TSS proximal transcribed regions (Fig. S8A and B). Notably, rs319824241 (*P* = 1.308 × 10^−13^) was identified as an independent variant associated with all three traits. Colocalization analysis of the GWAS summary statistics for three traits, performed using the “coloc” R package, demonstrated a strong colocalization signal for rs319824241 between SPPMOT and SPTACOIL (PP4 = 0.992), indicating the common regulatory mechanism between these traits. This variant is situated in an intergenic region, with the nearest gene being *ENSSSCG00000040707*.

To explore the regulatory mechanisms underlying this QTL, we extended our analysis by conducting colocalization using a 1 Mb region flanking the lead SNP rs319824241 and eQTL mapping summary statistics. This analysis identified 4,443 gene-tissue-trait pairs, of which 68 significant variant-gene-tissue associations (PP4 > 0.9) were identified (Table S7–S9 and Fig. 3D). Notably, rs319824241 displayed the strongest colocalization signal with the *DCAF12* gene (rs341819301, intron variant, in high LD (*r*^2^ = 0.825) with rs319824241) in muscle tissue across multiple semen quality traits, including SPPMOT, SPDCD, and SPTACOIL. The prominent colocalization signal observed in muscle tissue can be attributed to the larger sample size (1,321 samples) in the pigGTEx Atlas, which increases statistical power. In contrast, the relatively smaller sample size for testis (184 samples) may reduce the detection sensitivity, potentially explaining the mismatch between the colocalized tissue and semen quality traits. Additionally, rs341819301 significantly modulates *DCAF12* expression and is in moderate to high LD with the top eQTL (rs318690665, *r*^2^ = 0.751) of *DCAF12* (Fig. 3D and Fig. S9). And genotypic variations at rs341819301 were found to significantly influence the phenotypic variation of SPPMOT, SPDCD, and SPTACOIL (Fig. S10).

In order to further investigate the regulatory effects of rs341819301, chromatin state data were obtained from 14 tissues in the FAANG project to examine the influence of regulatory elements [23]. Enrichment analysis of rs341819301 revealed that it was most strongly enriched in the Bivalent/poised TSS (TssBiv) state, observed in 12 tissues, while it was sparsely enriched in the Repressed polycomb (Repr) state, found only in the spleen and cortex (Fig. S11). These findings suggest that rs341819301 likely regulates *DCAF12* expression by suppressing its transcriptional activity.

Moreover, phenome-wide association study (pheWAS) results revealed a significant association between rs341819301 and reproductive traits, including the number of teats and the number born of healthy pigs (Fig. S12 and Table S10). According to the pigGTEx resource, *DCAF12* is differentially expressed across tissues, with particularly high expression observed in the testis (Fig. S13). Knockdown of *DCAF12* in *Drosophila* testicular germline cells has been shown to reduce fertility [38]. Knockout of *DCAF12* in mice impairs spermatogenesis by promoting the ubiquitination and degradation of the MOV10 RNA helicase, thus disrupting RNA metabolism and gene expression, and ultimately reducing the production of mature sperm [39]. These findings collectively suggest that *DCAF12* may play a crucial role in regulating semen quality and fertility in boars.

### Integrative analysis highlights potential genes involved in SPMOT

To investigate the potential regulatory mechanisms underlying semen quality traits, we integrated pigGTEx resources and utilized integrative approaches to uncover the genetic basis of semen quality traits.

We first conducted TWAS for each trait using S-PrediXcan to uncover the potential regulatory mechanisms of 11 semen quality traits. The results revealed 58,162 gene-tissue-trait pairs associations for each trait. After Bonferroni correction, the number of significant genes in each tissue for SEMVOL, SPCON, SPDCD, SPDMR, SPFCOUNT, SPMOT, SPNR, SPPCD, SPPMOT, SPTABEND, and SPTACOIL was 58, 38, 56, 82, 26, 38, 52, 63, 71, 37, and 60, respectively (Fig. S14). In total, 581 significant gene-tissue-trait associations were identified, spanning 11 semen quality traits across 34 tissues. It is worth noting that muscle tissue exhibited the highest number of significant gene-tissue associations across multiple traits (such as SPPMOT, SPTACOIL, and SPNR), likely due to the larger sample size of muscle tissue in the pigGTEx resources.

**Fig. 4:**
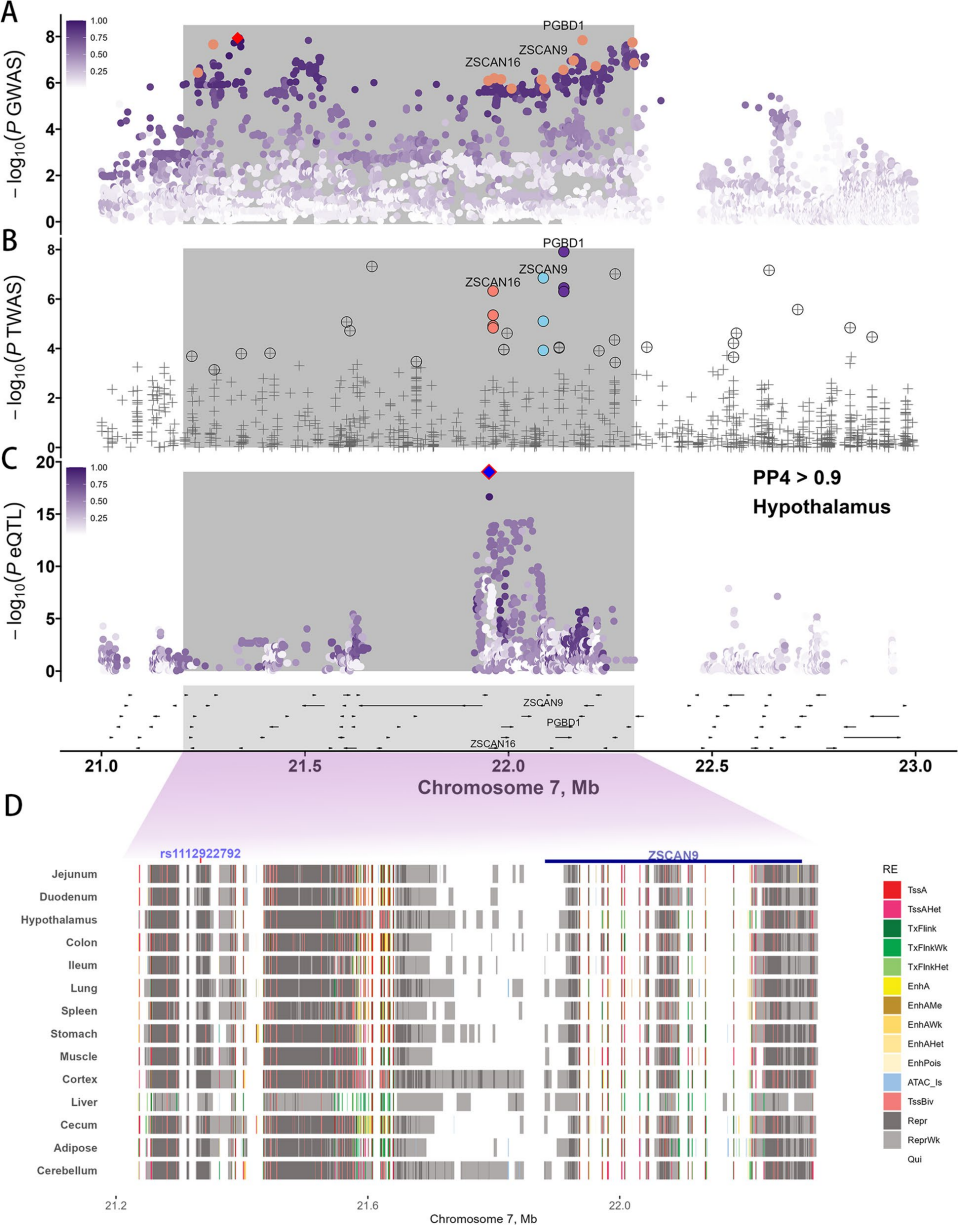
The regional functional analysis for SPMOT on chromosome 7. **A** The GWAS of independent variant rs1112922792 (red diamond point) with extending up and downstream 1 Mb. The red diamond shaped point represents the independent variant rs1112922792. The orange dots represent SNPs in strong linkage disequilibrium (*r*^2^ > 0.8) with the independent variants. The color bar represents the linkage disequilibrium between the independent variant and the other variants. **B** The single-tissue TWAS results. The grey crosses represent genes associated with SPMOT. The crossed circles indicate gene-phenotype pairs that reach the significance threshold (Bonferroni corrected). The orange dots represent the *ZSCAN16* associated with SPMOT in Oocyte, Testis, Intestine, and Blood tissues. The skyblue dots represent the *ZSCAN9* associated with SPMOT in Muscle, Adipose, and Intestine tissues. The purple dots represent the *PGBD1* associated with SPMOT in Ovary, Uterus, and Muscle tissues. **C** The eQTL mapping results for *ZSCAN16* in hypothalamus tissue. The colors correspond to the LD (*r*^2^) between independent variant and other variants. The blue diamond point represents the lead eQTL of *ZSCAN16* in hypothalamus. The red diamond point represents the Colocalization loci rs1109397868. The bottom panel displays the gene positioning, with arrows indicating the direction of gene expression. **D** The chromatin states of each tissue in the candidate genomic regions identified in the GWAS results for SPMOT. The key regulatory SNP rs1112922792 is highlighted above the red rectangle, while the candidate gene *ZSCAN9* is indicated above the blue rectangle. The colors correspond to regulatory element type

Furthermore, we integrated single-trait GWAS, TWAS, and Colocalization to explore the genetic mechanisms underlying SPMOT. Firstly, we conducted GWAS for the SPMOT trait using a single-trait repeatability model, which revealed significant association signals on SSC7 (Fig. S15). Through conditional analysis using GCTA-COJO, rs1112922792 was identified as an independent variant with the highest −log_10_*P* value (Fig. S16). Annotation of this SNP indicated that it is located downstream of the *PRSS16* gene. To refine the QTL region, we identified a 1.1 Mb segment (21,200,707–22,308,871) in strong LD (*r*^2^ > 0.8) with the lead SNP (Fig. 4A). Further annotation of the SNPs within this QTL shows that most of the SNPs are located in non-coding regions (Fig. S17B), with functional annotation revealing that SNPs are most enriched in transcription start site (TSS)-proximal transcribed regions (Fig. S17A). Fourteen potential candidate genes within the QTL include: *PRSS16*, *ZSCAN31*, *PGBD1*, *ZSCAN12*, *SCAN3*, *ZSCAN23*, *ZSCAN26*, *H2AC12*, *ZSCAN16*, *ZKSCAN8*, *ZSCAN9*, *ZNF165*, *ZNF389*, and *ZKSCAN4*. To further narrow down the candidate genes, gene-based association analysis was performed using MAGMA, which identified 25,286 gene-trait associations (Fig. S18 and Table S11). Ten genes were found to be significantly associated with SPMOT (*P* < 3.95 × 10^–^^5^) and were consistently significant in both variant-based and gene-based association studies: *PRSS16*, *ENSSSCG00000048452*, *ZSCAN16*, *ZKSCAN8*, *ENSSSCG00000021069*, *ZSCAN9*, *PGBD1*, *ZSCAN31*, *ZSCAN12*, and *GPX5*.

To investigate the molecular mechanisms through which variations in gene expression effect SPMOT, We performed single-tissue TWAS using S-PrediXcan. Our analysis uncovered 58,163 gene-tissue-trait associations, of which 38 were found to be statistically significant following Bonferroni correction (Fig. 4B and Table S12). Notably, the candidate genes identified through both variant-based and gene-based association studies also showed strong associations in the TWAS. Specifically, *ZSCAN16* (in Oocyte, Testis, Intestine, and Blood tissues), *ZSCAN9* (in Muscle, Adipose, and Intestine tissues), and *PGBD1* (in Ovary, Uterus, and Muscle tissues) exhibited significant signals in the TWAS, overlapping with the candidate genes identified in previous analyses.

To further explore the regulatory relationship between SNPs and genes, and phenotypes, we performed colocalization analysis using the “coloc” R package. The lead SNP (rs1112922792, downstream gene variant) colocalized with 2,826 gene-tissue-trait associations between the SPMOT GWAS summary and the eQTL summary statistics. Of these, nine variant-gene-tissue pairs were identified as statistically significant (PP4 > 0.9, Table S13), with two genes being consistently identified across GWAS, gene-based GWAS, and TWAS analyses (Fig. S19). Specifically, *ZSCAN16* in the hypothalamus (rs1109397868, PP4 = 0.99, Fig. 4C) and *ZSCAN9* in the uterus (rs322211455, PP4 = 0.91, Fig. S20) was demonstrated significant colocalization with the QTLs of lead SNP. Notably, rs322211455, which is in high linkage disequilibrium (*r*^2^ = 0.82) with the lead SNP, is located in an intergenic region. This variant was found to significantly modulate the expression of *ZSCAN9* (Fig. S21), and different genotypes at this variant were found to have a notable impact on sperm motility, aligning with the expression changes observed with the lead SNP (Fig. S22). Additionally, rs322211455 resides within a Repr state in 13 tissues (including muscle, adipose, and lung, etc.) and the ReprWk state in liver (Fig. 4D), suggesting its potential involvement in the regulatory mechanism of SPMOT by suppressing the expression of *ZSCAN9*.

Finally, we conducted a pheWAS for rs322211455 across 260 traits (Fig. S23 and Table S14), revealing a significant association with sperm abnormality rate (DRP), underscoring the potential importance of this locus in the genetic architecture of semen quality. Additionally, we compared the expression patterns of *ZSCAN9* across various tissues and found that it was most highly expressed in the hypothalamus, testis, and pituitary gland (Fig. S24). Notably, *ZSCAN9* expression in the human placenta was influenced by several eSNPs, which affect the methylation levels of genes escaping X chromosome inactivation [40]. These findings indicate that *ZSCAN9* likely regulates the physiological function of the gonads through the hypothalamic-pituitary–gonadal axis, which may also explain the strong colocalization signal observed for rs322211455 in the uterus.

## Discussion

This study provides a comprehensive exploration of the genetic architecture underlying semen quality traits in Duroc boars, providing key insights into the genetic regulation of these critical reproductive traits. Numerous GWAS have identified thousands of variants associated with various semen quality traits in pigs [17, 41]. With the rapid advancement of the post-GWAS era, the focus has shifted from identifying genetic regions associated with semen quality to pinpointing genetic variants that regulate gene expression and the target genes driving these signals [42], thereby advancing our understanding of the regulatory mechanisms in semen quality. In this study, we conducted single-trait GWAS for 11 semen quality traits to identify candidate genes. By leveraging the FAANG and PigGTEx resources and employing TWAS and colocalization, we further investigated the role of gene expression in mediating phenotypes and variations, underlying potential regulatory mechanisms. This integrative approach not only deepens our understanding of the genetic basis of semen quality but also provides new insights into the transcriptional regulatory mechanisms volved.

The summary statistics of the semen quality phenotypes indicated that sperm morphological abnormalities such as SPDCD, SPPCD, SPTACOIL, and SPTABEND exhibited high phenotypic variability. These traits primarily reflect the presence of distal and proximal cytoplasmic droplets and the degree of tail bending. The percentage of aberrant sperm morphology was minimal in the majority of samples, with trait values predominantly concentrated in the 0–1 range. A limited number of individuals displayed significant anomalies, leading to a skewed distribution and high phenotypic variability. In addition, the accuracy of detecting slight morphological differences may be influenced by the sensitivity and parameter settings of the CASA system. Environmental factors could also exacerbate the variability of these traits. Importantly, our statistical model corrected for potential confounding factors to ensure the robustness of the results. From a genetic perspective, the traits are likely regulated by polygenes, and the historically low selection pressure may have preserved a wide range of genetic variation. Figure 2A further corroborates these findings by identifying significant loci associated with sperm morphological abnormalities across multiple QTLs on several chromosomes, highlighting their complex genetic architecture. In our study, the estimated heritability of boar semen quality traits ranged from 0.095 to 0.343, which is generally consistent with the previously reported range of 0.06 to 0.39 [16, 17, 41]. This consistency supports the accuracy and reliability of our phenotypic measurements and data processing. Repeatability estimates ranged from 0.284 to 0.586, indicating that in addition to genetic effects, permanent environmental factors (such as housing conditions, management routines, and individual boar characteristics) also contribute substantially to the phenotypic variance. The moderate repeatability and low heritability of some traits may also reflect gene-by-environment interaction (G × E), the effects of which may weaken the effect of genetic variation.

Additionally, although no direct association between the *DCAF12* region and semen quality phenotypes was observed in our single-trait GWAS, the integration of PigGTEx resources and the use of colocalization analysis, revealed that semen quality traits (SPPMOT, SPDCD, and SPTACOIL) colocalized with the *DCAF12* (rs341819301). This suggests that *DCAF12* may influence multiple semen quality traits through transcriptional regulation and emphasizing the importance of transcriptional regulation in understanding phenotypic variation. Supporting this hypothesis, Cauchi demonstrated that knockdown of *DCAD12* in *Drosophila* led to reduced fertility and decreased sperm counts in germ cells of the testes [38]. Similarly, Lidak et al. [39] generated *DCAF12*-knockout mice and showed that *DCAF12*-mediated degradation of MOV10 affects spermatogenesis, resulting in fewer mature sperm produced in the testes. Moreover, *DCAF12* is found to be highly expressed in pigs (Fig. S11), further supporting its potential regulatory role in porcine semen quality.

We also identified other candidate genes, including *ZSCAN9* and *ZSCAN16*, involved in semen quality regulation. TWAS revealed that *ZSCAN9*, *ZSCAN16*, and *PGBD1* were significantly associated with SPMOT across multiple tissues, such as the testis and oocyte. Colocalization analysis further demonstrated strong signals between *ZSCAN9* (expressed in the uterus) and *ZSCAN16* (expressed in the hypothalamus) with SPMOT. Previous studies have shown that *ZSCAN9* expression is regulated by multiple eSNPs (experimentally validated: rs1150707), some of which also influence the methylation levels of genes that variably escape X-chromosome inactivation in humans [40]. In addition, Kikas et al. [43] identified several significant *cis*-eQTLs in the placenta that regulate the expression of *RPL9*, *ZSCAN9*, and *ERAP2*, implicating *ZSCAN9* in placental development. Notably, in pigs, *ZSCAN9* is highly expressed in the testis, hypothalamus, and pituitary, underscoring its potential involvement in semen quality regulation via the hypothalamic-pituitary-gonadal (HPG) axis [44].

Despite the valuable insights gained from this study, several limitations must be considered. First, although we integrated transcriptomic data from the PigGTEx resource, the sample size for certain tissues may still be insufficient to fully capture the genetic diversity of semen quality traits. Expanding the sample set could further enhance the power and reliability of our findings. Second, while TWAS and colocalization analysis revealed promising candidate genes, their precise functional roles in regulating semen quality require further validation through experimental studies. Additionally, although TWAS and colocalization have enhanced our understanding of semen quality regulation, they are based on existing reference data, which may not capture all genetic variants affecting gene expression in reproductive tissues. Future studies with more comprehensive transcriptomic datasets could provide a comprehensive insight on the genetic regulation of semen quality in pigs.

## Conclusion

In summary, integrating large-scale population data from GWAS and pigGTEx resources through an integrative analysis offers a promising approach to uncovering the genetic architecture of complex traits, providing a deeper understanding of complex traits. Through integrative analysis, we found that *DCAF16* and *ZSCAN9* are candidate genes with high reliability in semen quality for boars.

## Supplementary Information

Additional file 1: Table S1. Descriptive statistics, heritability, and repeatability estimates for 11 semen traits in duroc boars. Table S2. The lambda for 11 semen traits. Table S3. The independent variants for 11 semen traits through GCTA-COJO conditional analysis. Table S4. The summary statistics for validation population in Duroc boars. Table S5. The significant enrichment analysis of gene ontology for 11 semen traits. Table S6. The significant enrichment analysis of KEGG pathway for 11 semen traits. Table S7. The significant colocalization genes with PP4 > 0.9 in SPDCD. Table S8. Significant colocalization genes with PP4 > 0.9 in SPPMOT. Table S9. Significant colocalization genes with PP4 > 0.9 in SPTACOIL. Table S10. The PheWAS analysis of rs341819301. Table S11. The significantly associated gene list from gene-based association analysis in SPMOT. Table S12. The significantly associated gene list from single-tissue TWAS analysis in SPMOT. Table S13. Significant colocalization genes with PP4 > 0.9 in SPMOT. Table S14. The PheWAS analysis of rs322211455.

Additional file 2: Fig. S1. The histogram showing the distribution of traits measurements across all individuals in the study population. Fig. S2. The histogram of the number of ejaculation records per sire. Fig. S3. The accuracy of genotype imputation. Fig. S4. The heatmap of kinship relationships among individuals. Fig. S5. The PCA before (left) and after (right) imputation using the Pig Genotype Reference Panel (PGRP). Fig. S6. The *π*1 statistic for discovery in validation population or validation in discovery population. Fig. S7. The significant enrichment of GO term and KEGG pathway for individual GWAS in all semen quality traits. Fig. S8. Functional annotation and variant classification for SPPMOT, SPDCD, and SPTACOIL. Fig. S9. The most significant colocalization signal (rs341819301) of *DCAF12* and top eQTL (rs318690665) of *DCAF12* significantly contribute to the regulation of *DCAF12* expression levels. Fig. S10. The effect of the three different genotypes of rs341819301 on the phenotypic variation of SPTACOIL, SPPMOT, and SPDCD. Fig. S11. The chromatin states of each tissue in the candidate QTL region identified in the GWAS results for SPPMOT, SPTACOIL, and SPDCD are shown. Fig. S12. The PheWAS of rs341819301 in pig. Fig. S13. The expression level of *DCAF12* in multiple tissues. Fig. S14. The number of significant gene-tissue-phenotype pairs identified in each tissue for TWAS of each trait. Fig. S15. The Manhattan plot for SPMOT GWAS. Fig. S16. The conditional GWAS regional Manhattan plot for rs1112922792 in SPMOT. Fig. S17. Functional annotation and variant classification for SPMOT. Fig. S18. The gene-based association analysis for SPMOT. Fig. S19. The upsetR summary of GWAS and post-GWAS in SPMOT. Fig. S20. The eQTL mapping results for *ZSCAN9* in uterus tissue. Fig. S21. The most significant colocalization signal (rs322211455) of *ZSCAN9* and top eQTL (rs1112922792) of *ZSCAN9* significantly contribute to the regulation of *ZSCAN9* expression levels. Fig. S22. The impact of the three genotypes of rs322211455 (the colocalization signal in *ZSCAN9*) and rs1112922792 (the lead SNP in the SPMOT GWAS) on the phenotypic variation of SPMOT. Fig. S23. The PheWAS of rs322211455 in pig. Fig. S24. The expression level of *ZSCAN9* in multiple tissues.

## Data Availability

The datasets supporting the conclusions of this article are included within the article and its additional files.

## Abbreviations

AI: Artificial insemination
eQTLs: Expression quantitative trait loci
FAANG: Functional Annotation of Animal Genomes
GO: Gene Ontology
GTEx: The Genotype-Tissue Expression
GWAS: Genome wide association studies
KEGG: Kyoto Encyclopedia of Genes and Genome
LD: Linkage disequilibrium
PGRP: Pig Genomics Reference Panel
pheWAS: Phenome-wide association study
QTLs: Quantitative trait loci
Repr: Repressed polycomb
SNP: Single nucleotide polymorphism
TSS: Transcription start site
TssBiv: Bivalent/poised TSS
TWAS: Transcriptome-wide association study
