# Supplementary material for: Integrative pigGTEx resource with GWAS reveals genetic mechanism underlying semen quality in boars
Source: J Anim Sci Biotechnol. 2025 Jul 21;16:105. doi: 10.1186/s40104-025-01237-2 (PMC12278574; doi:10.1186/s40104-025-01237-2)
Supplement: Supplementary file 2 — Additional file 2: Fig. S1. The histogram showing the distribution of traits measurements across all individuals in the study population. Fig. S2. The histogram of the number of ejaculation records per sire. Fig. S3. The accuracy of genotype imputation. Fig. S4. The heatmap of kinship relationships among individuals. Fig. S5. The PCA before (left) and after (right) imputation using the Pig Genotype Reference Panel (PGRP). Fig. S6. The π1 statistic for discovery in validation population or validation in discovery population. Fig. S7. The significant enrichment of GO term and KEGG pathway for individual GWAS in all semen quality traits. Fig. S8. Functional annotation and variant classification for SPPMOT, SPDCD, and SPTACOIL. Fig. S9. The most significant colocalization signal (rs341819301) of DCAF12 and top eQTL (rs318690665) of DCAF12 significantly contribute to the regulation of DCAF12 expression levels. Fig. S10. The effect of the three different genotypes of rs341819301 on the phenotypic variation of SPTACOIL, SPPMOT, and SPDCD. Fig. S11. The chromatin states of each tissue in the candidate QTL region identified in the GWAS results for SPPMOT, SPTACOIL, and SPDCD are shown. Fig. S12. The PheWAS of rs341819301 in pig. Fig. S13. The expression level of DCAF12 in multiple tissues. Fig. S14. The number of significant gene-tissue-phenotype pairs identified in each tissue for TWAS of each trait. Fig. S15. The Manhattan plot for SPMOT GWAS. Fig. S16. The conditional GWAS regional Manhattan plot for rs1112922792 in SPMOT. Fig. S17. Functional annotation and variant classification for SPMOT. Fig. S18. The gene-based association analysis for SPMOT. Fig. S19. The upsetR summary of GWAS and post-GWAS in SPMOT. Fig. S20. The eQTL mapping results for ZSCAN9 in uterus tissue. Fig. S21. The most significant colocalization signal (rs322211455) of ZSCAN9 and top eQTL (rs1112922792) of ZSCAN9 significantly contribute to the regulation of ZSCAN9 expression levels. Fig. S22. The impa [file 40104_2025_1237_MOESM2_ESM.docx]

**Additional file 2**


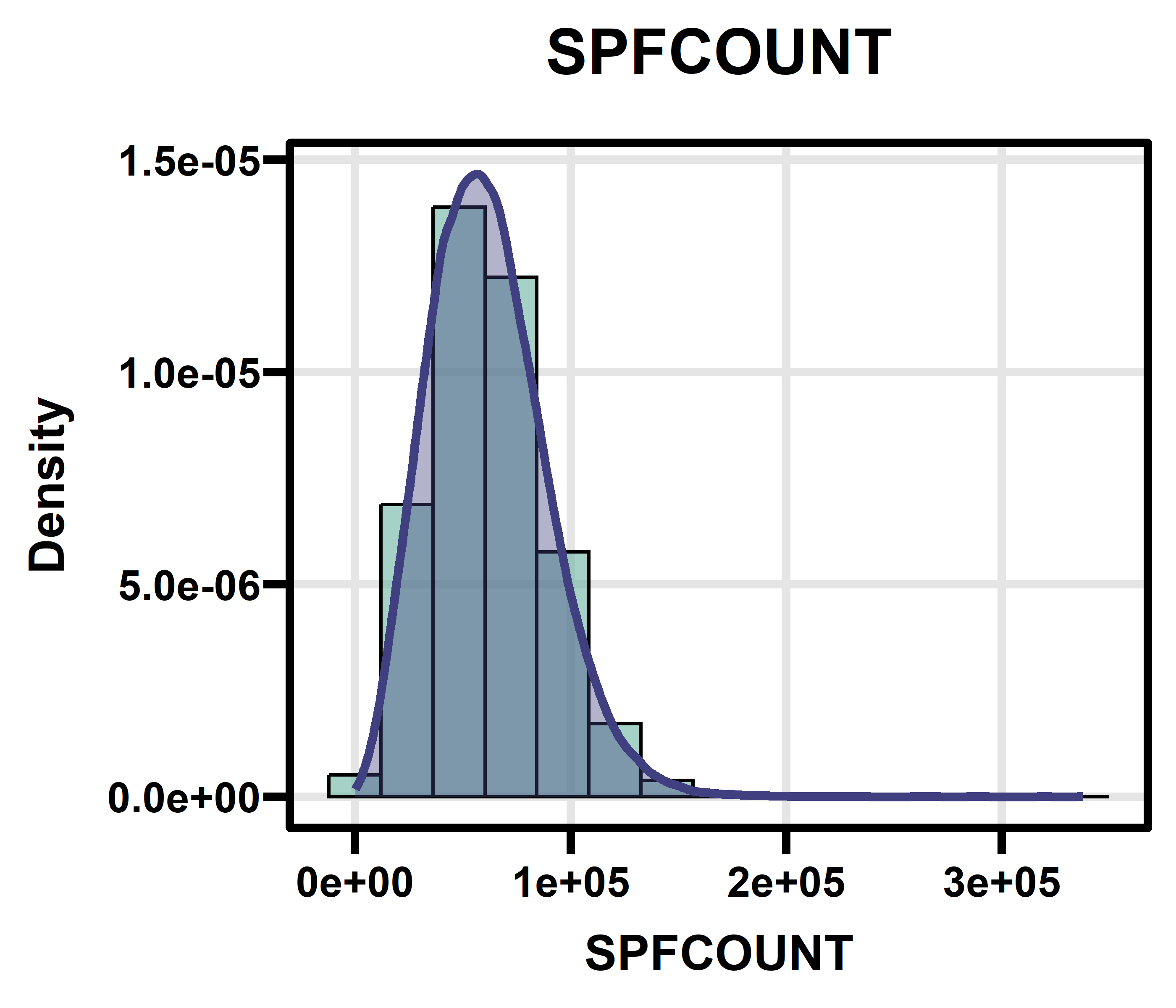

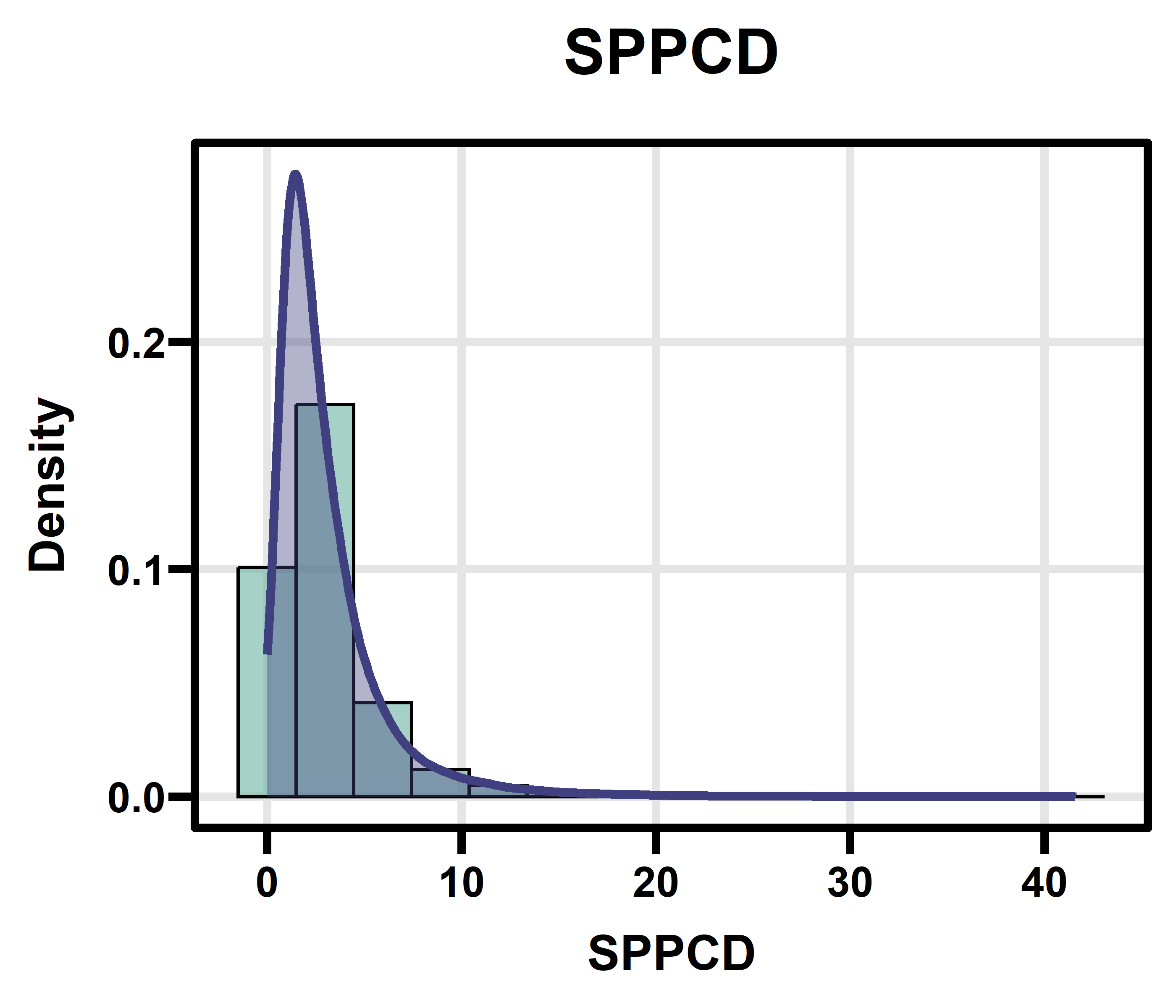

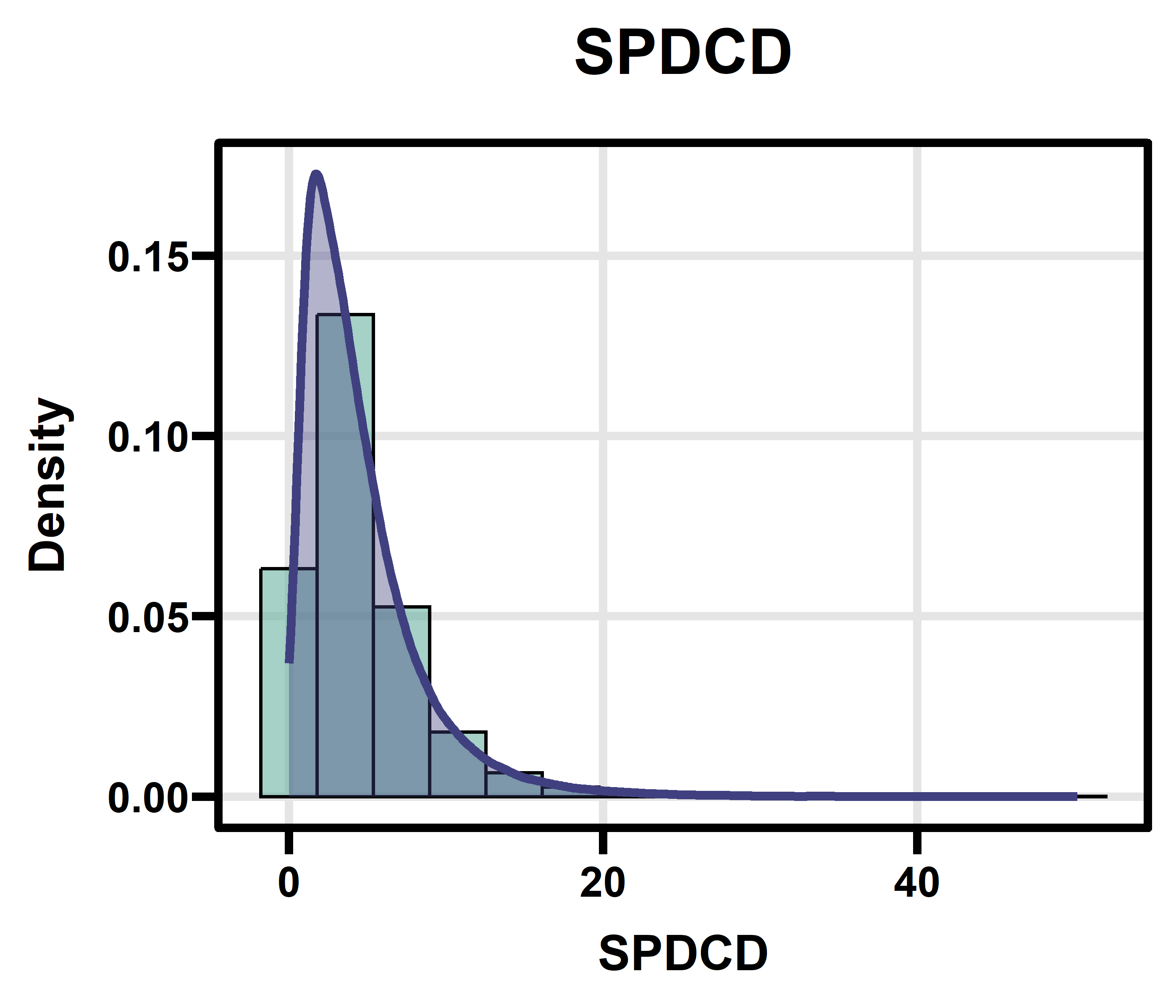

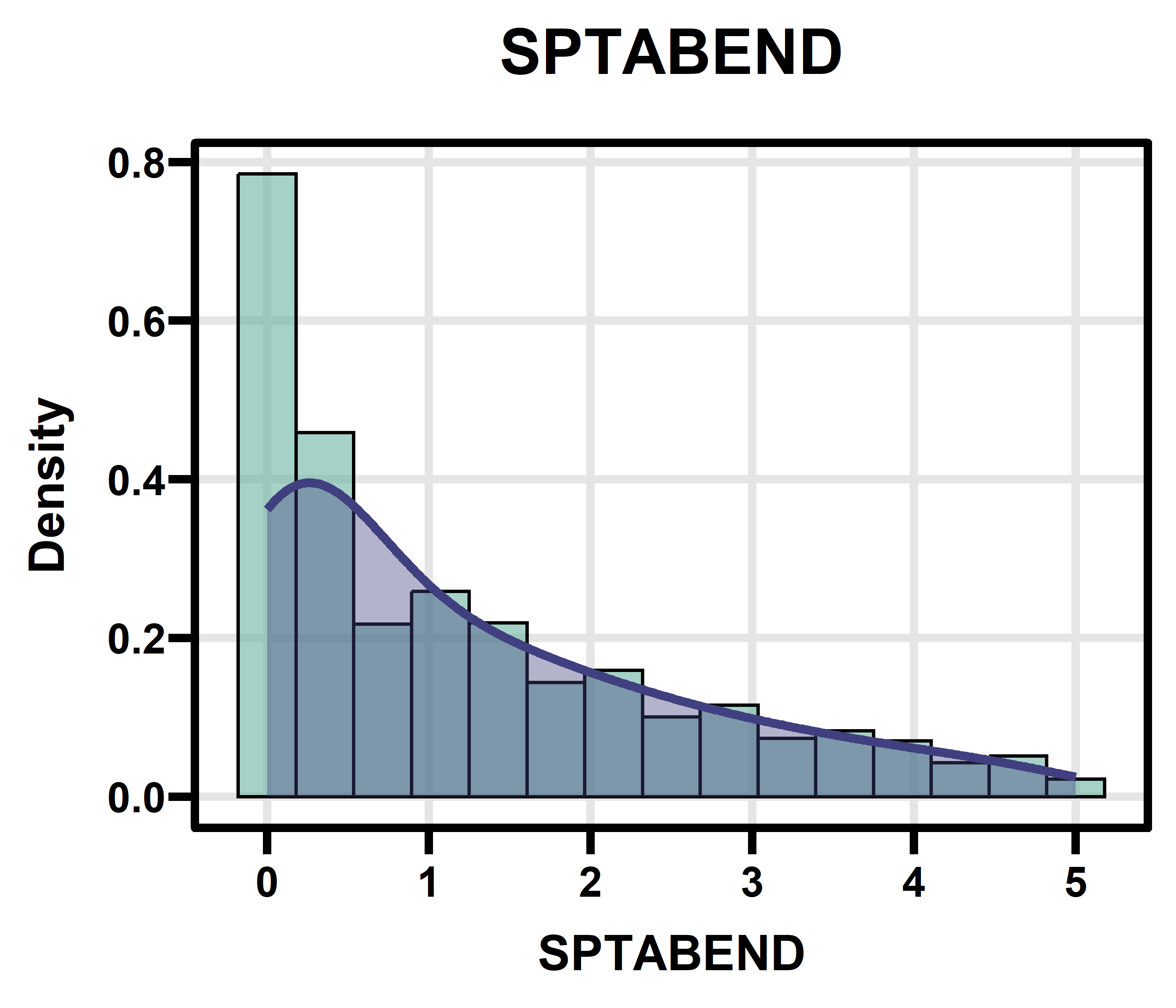

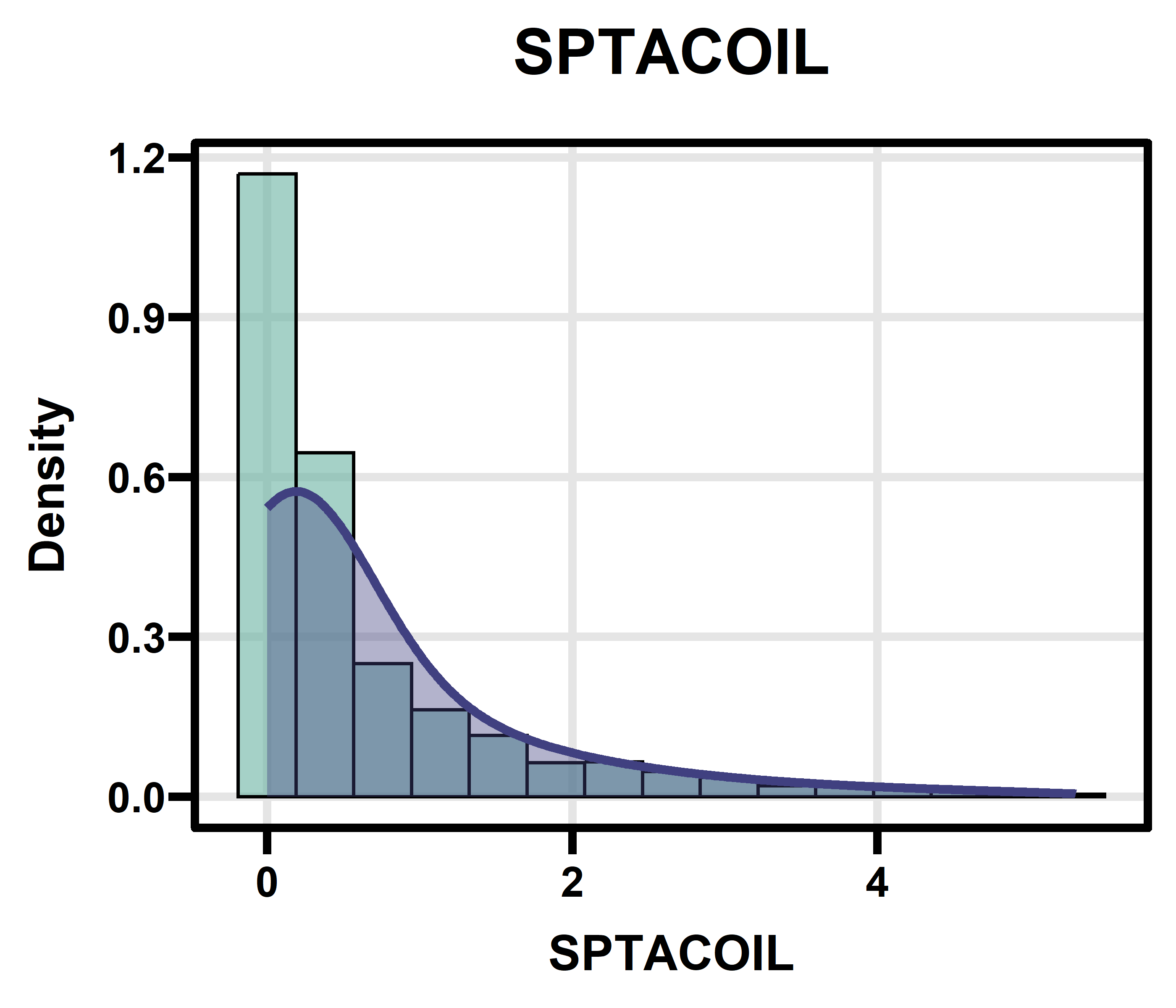

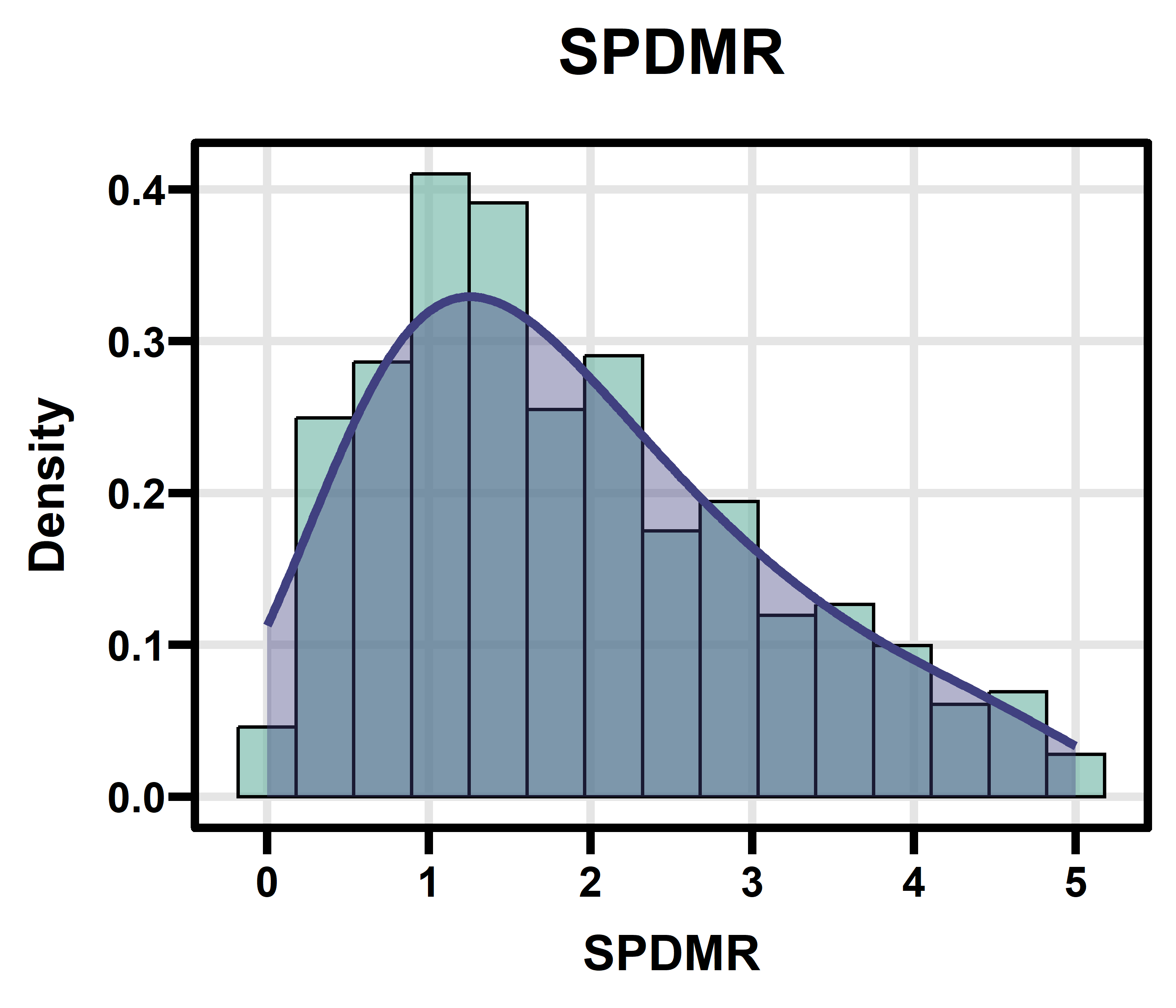

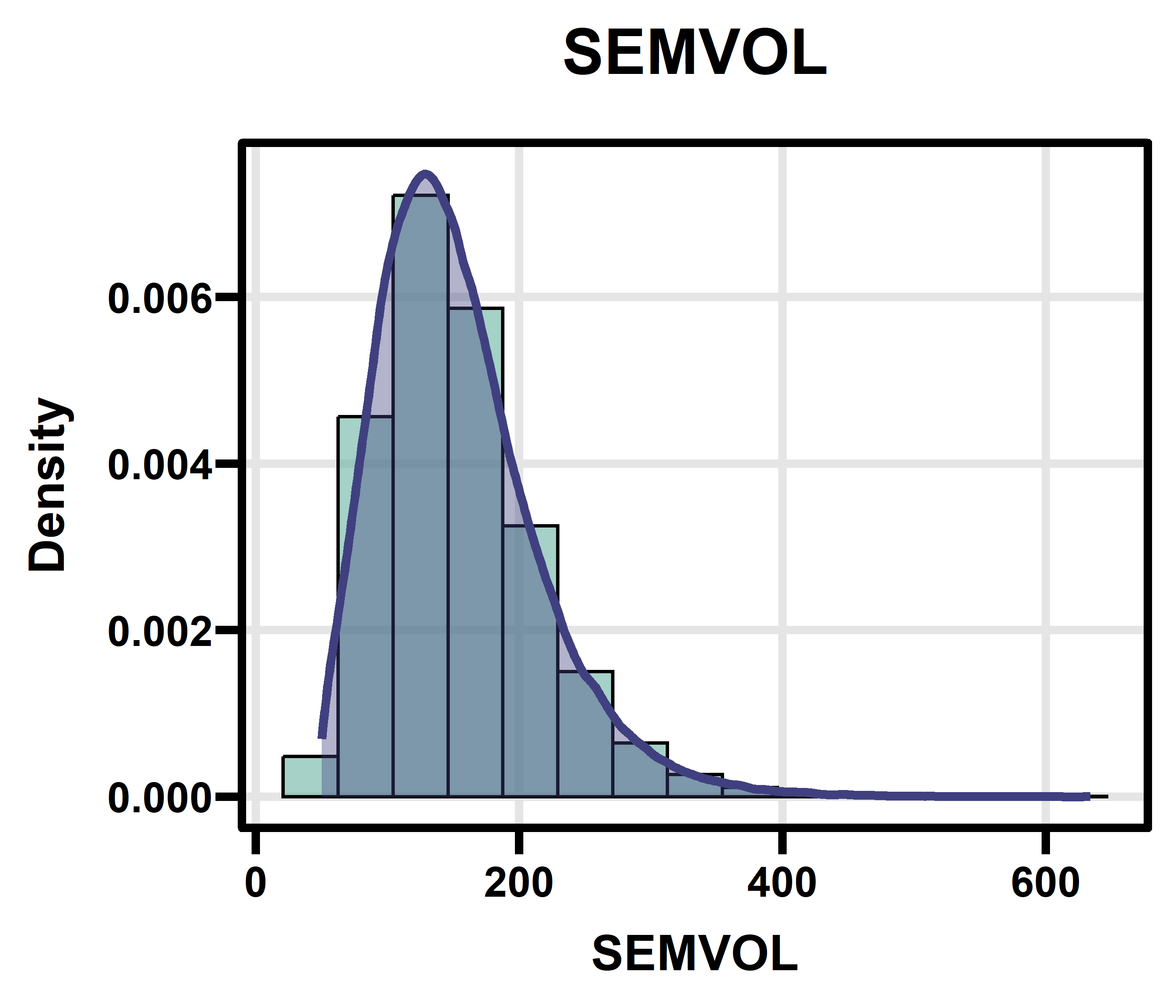
**8**
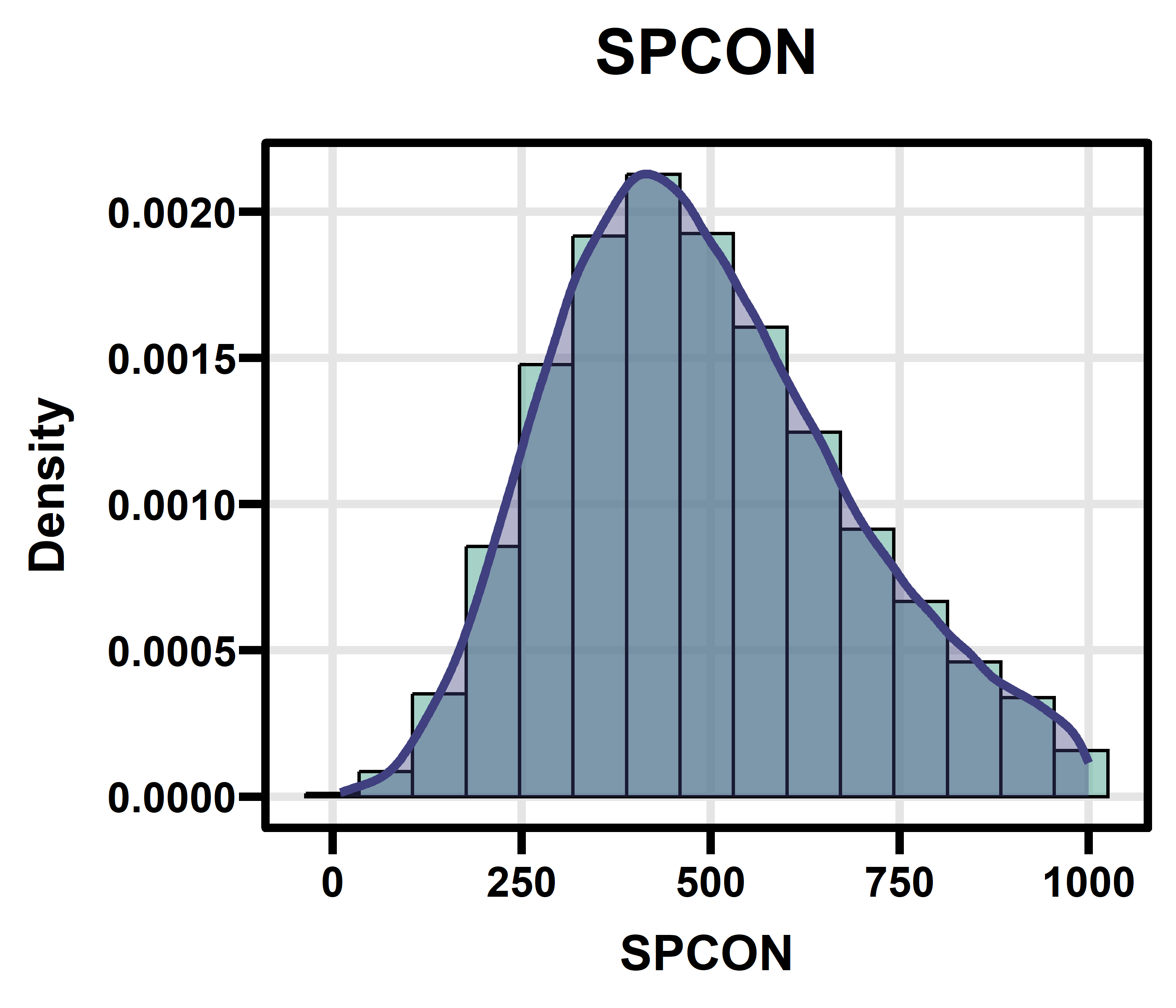

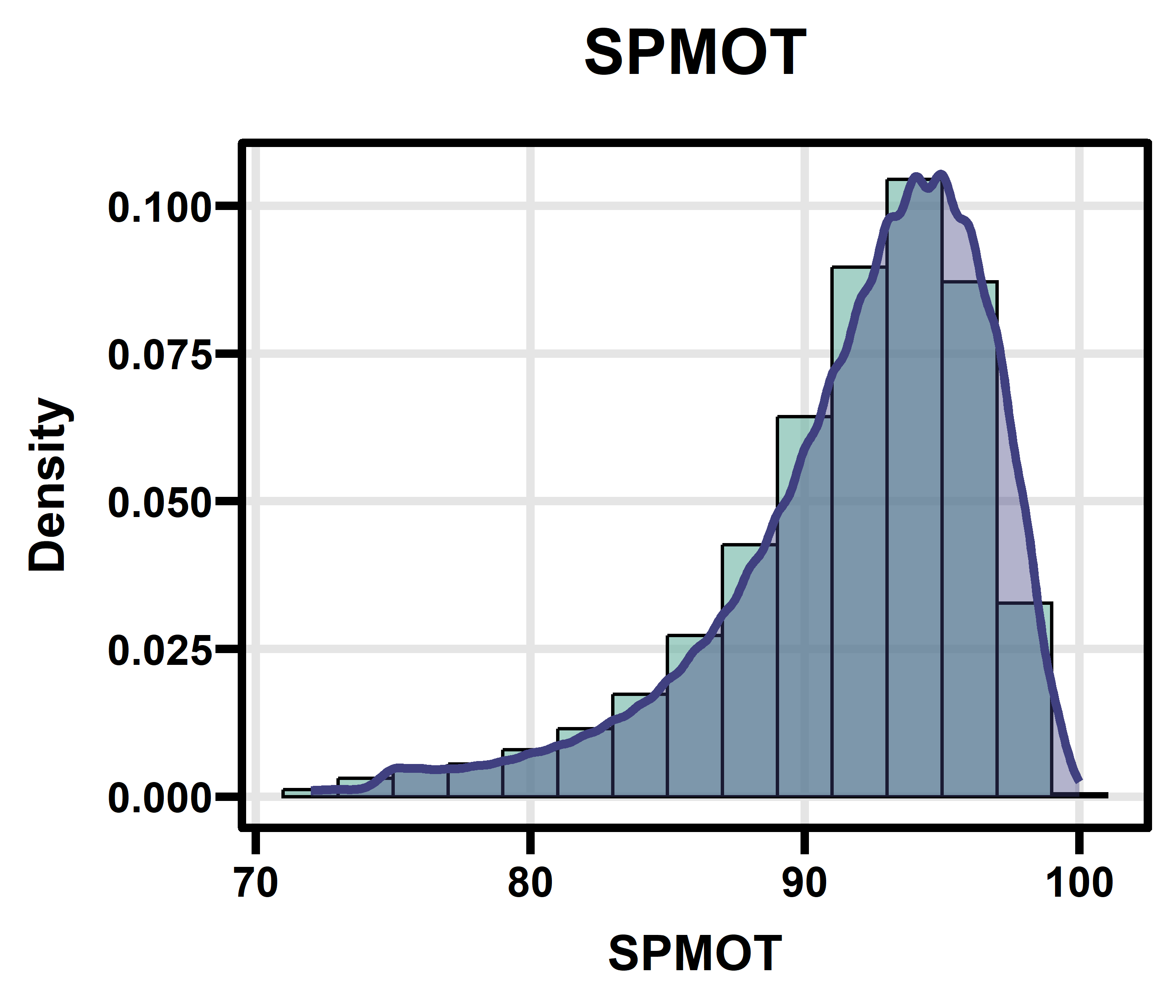

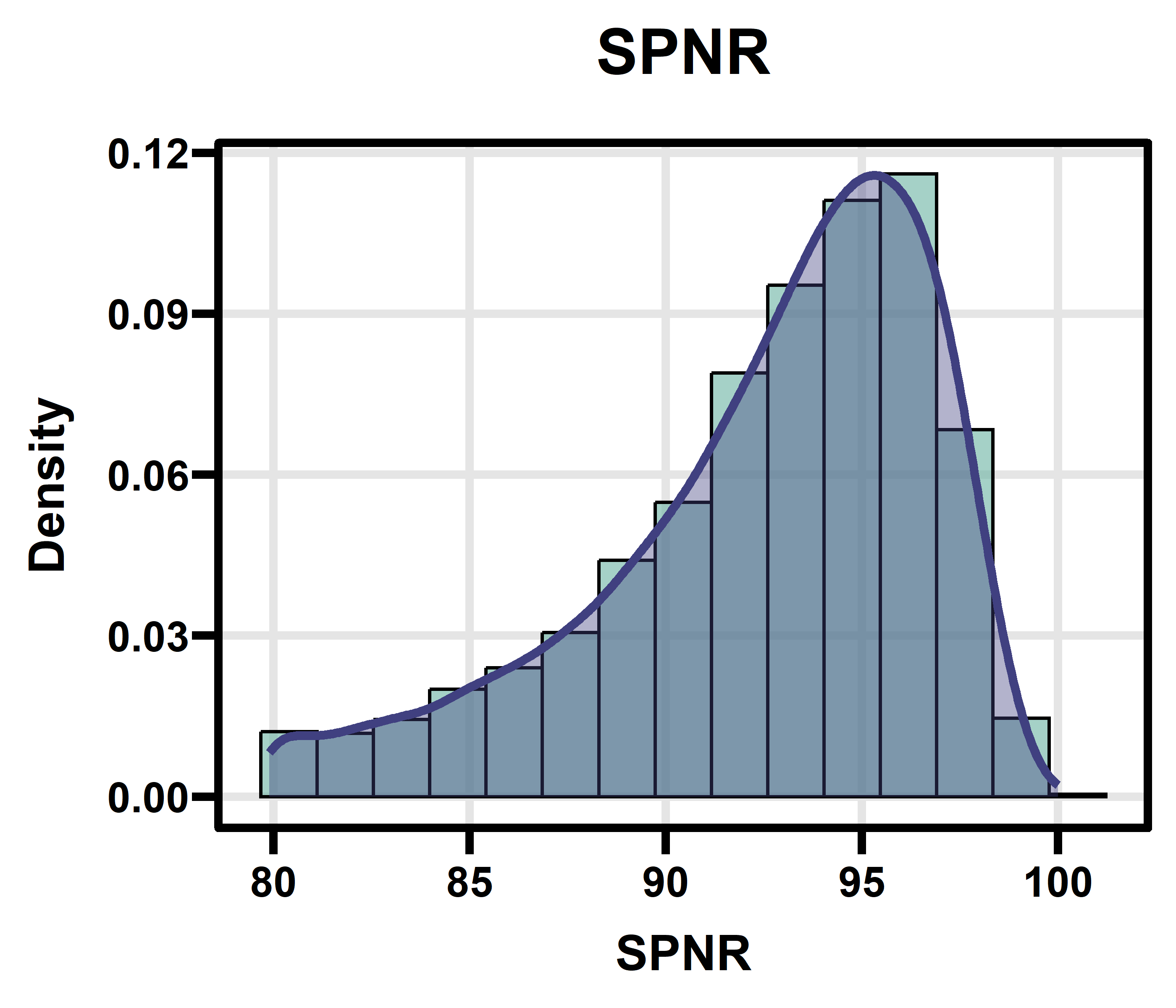
**
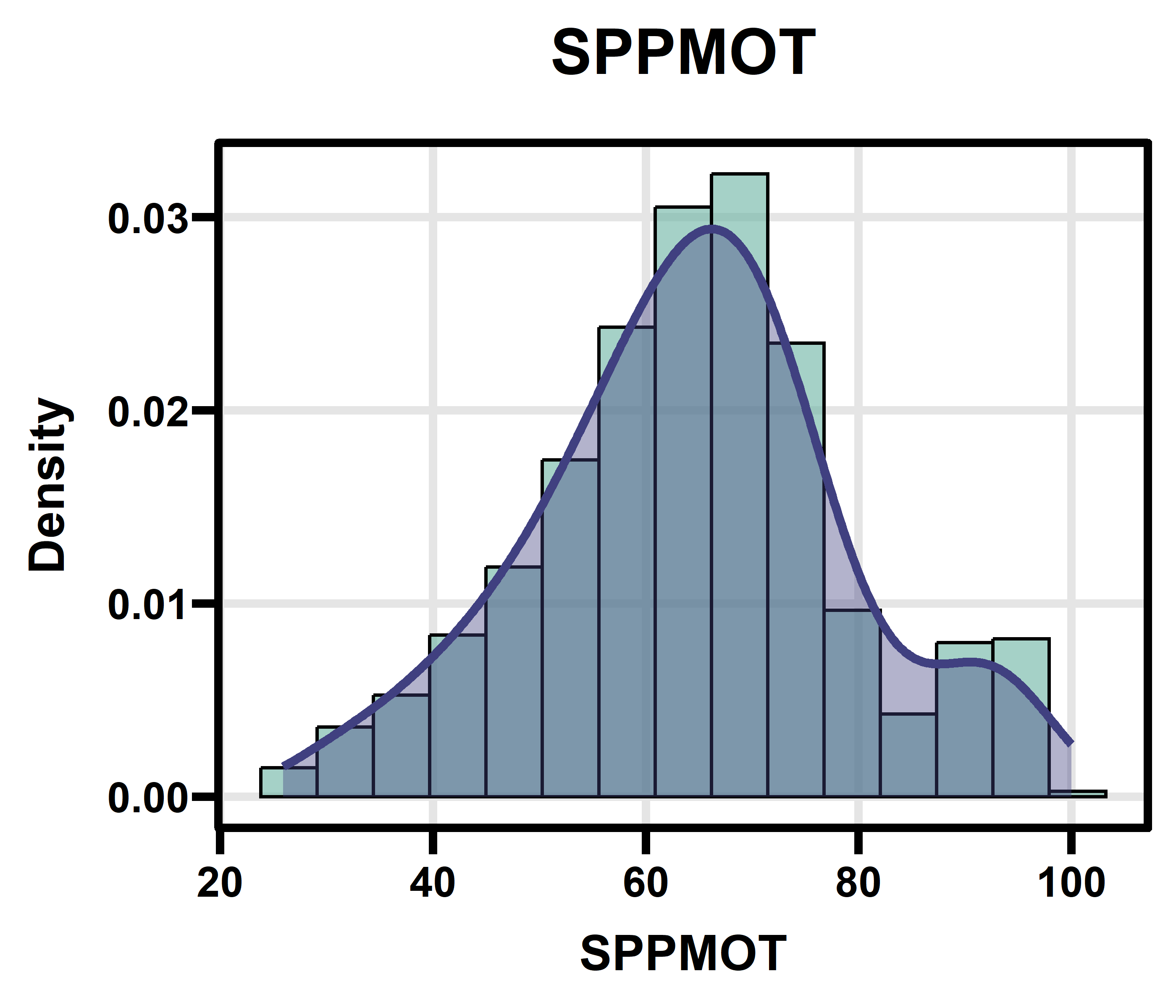
**

**Fig. S1** The histogram showing the distribution of trait measurements across all individuals in the study population


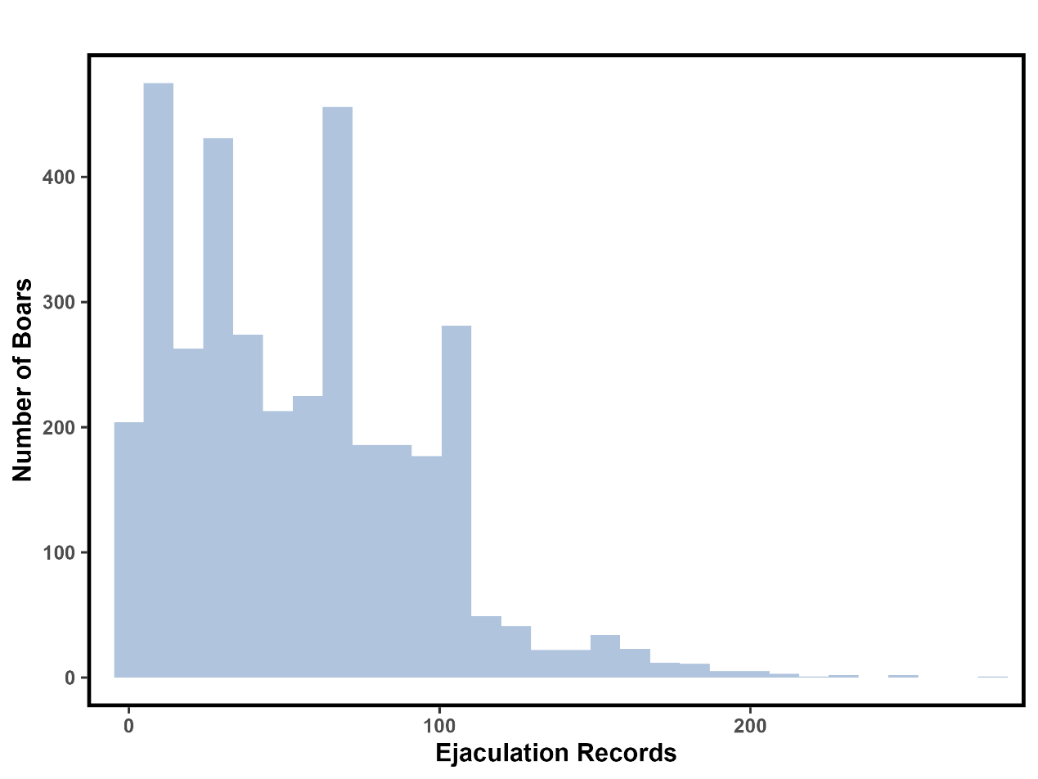


**Fig. S2** The histogram of the number of ejaculation records per sire


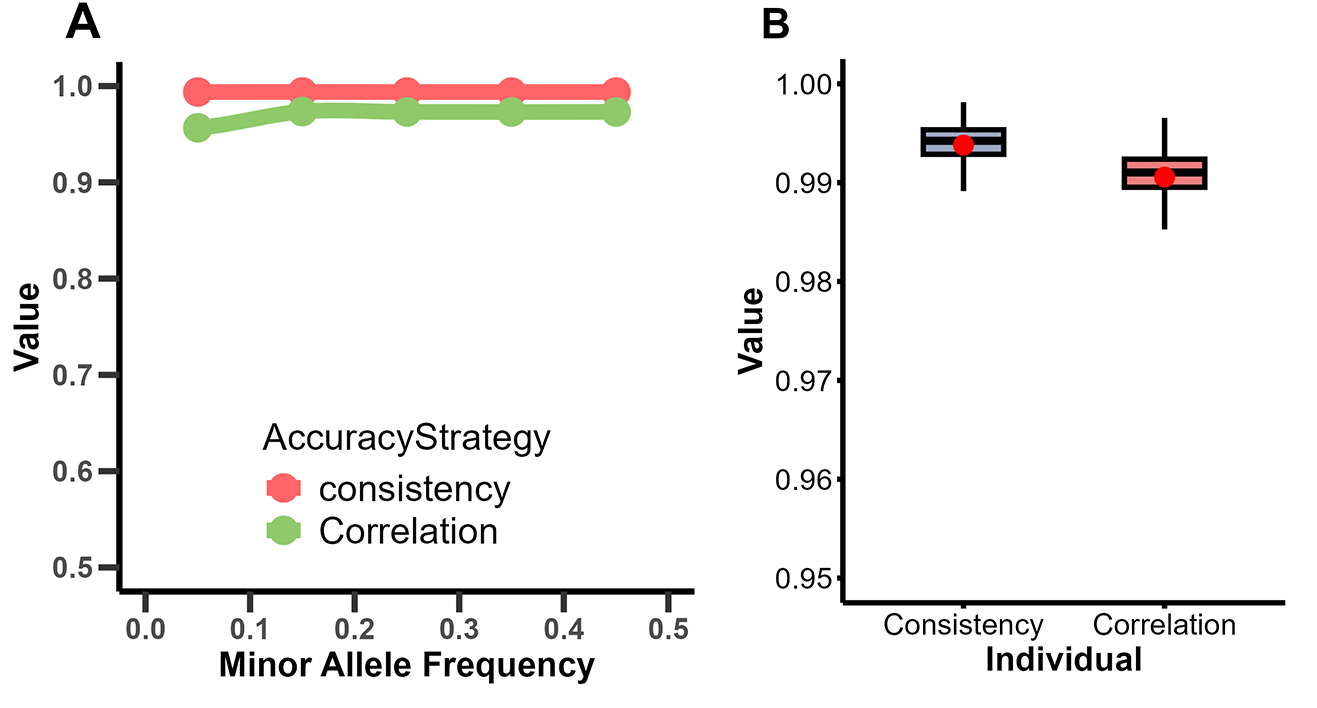


**Fig. S3** The accuracy of genotype imputation. **A** The distribution of Minor Allele Frequency (MAF) and imputation accuracy (genotype consistency and correlation).

**B** The performance of genotype imputation across all GWAS populations at the individual level


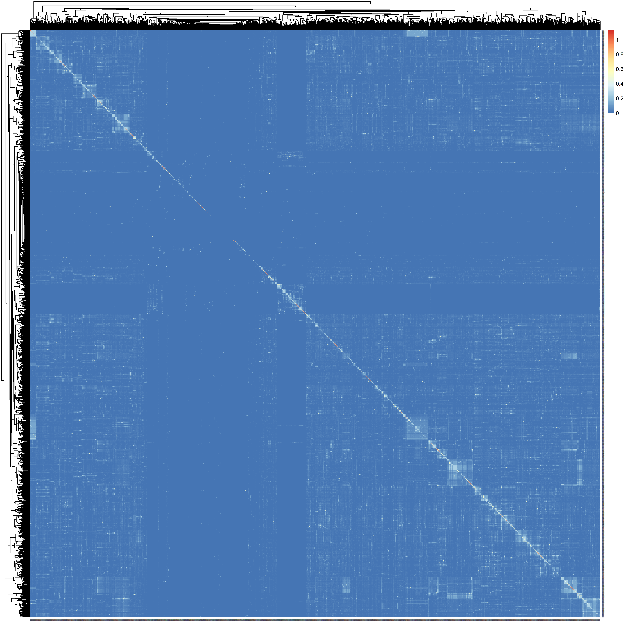

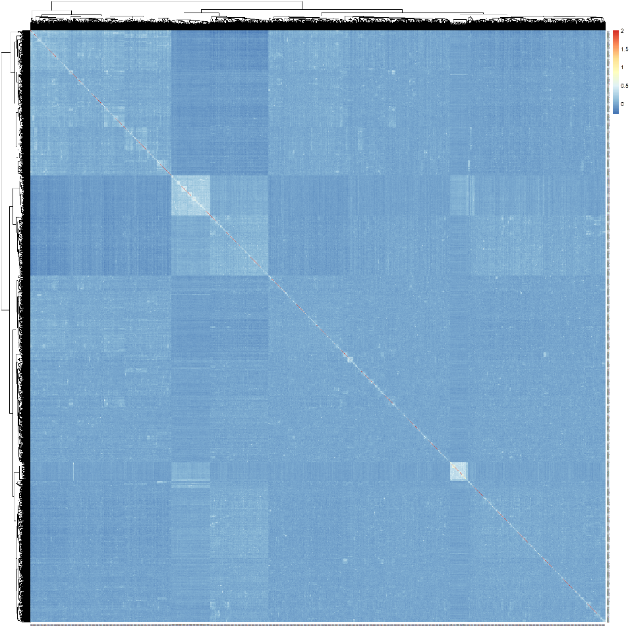


**Fig. S4** The heatmap of kinship relationships among individuals is presented, with the left side based on the A matrix and the right side based on the G matrix


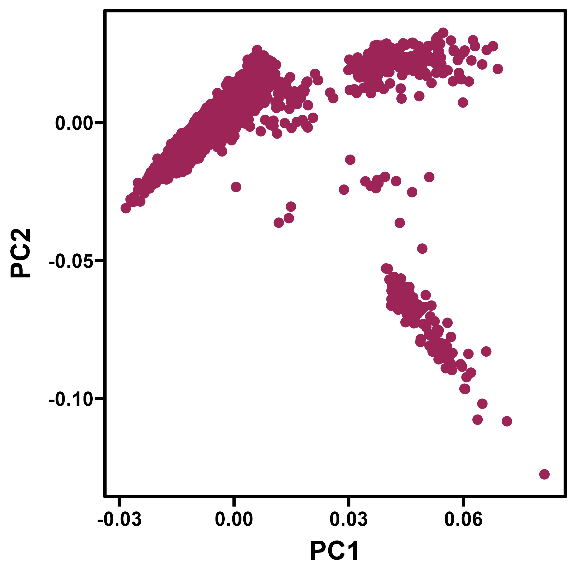

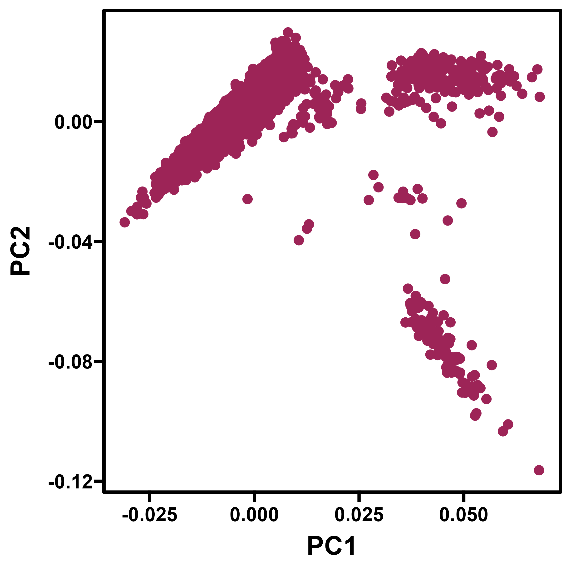


**Fig. S5** The PCA before (left) and after (right) imputation using the Pig Genotype Reference Panel (PGRP)


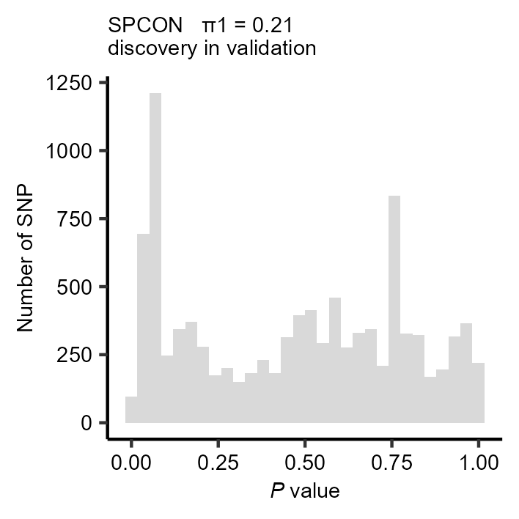

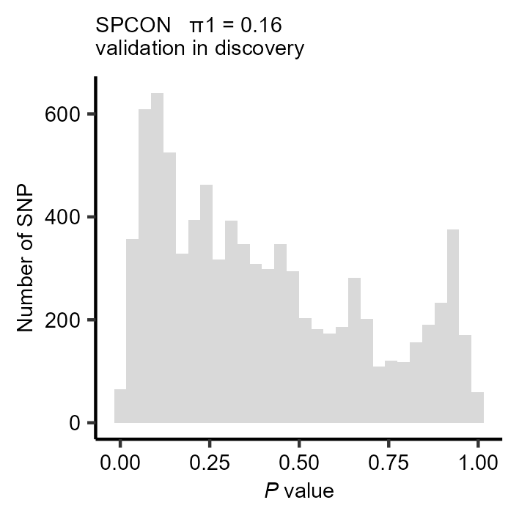

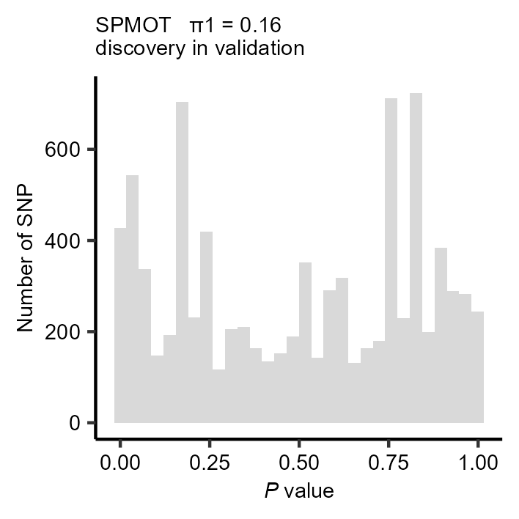

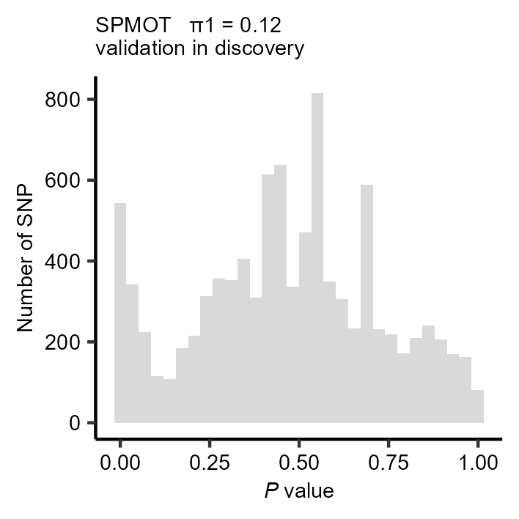


**Fig. S6** The $\pi1$ statistic for discovery in validation population or validation in discovery population. Left panel (discovery in validation): Shows the distribution of *P*-values for SNPs identified in the discovery population when tested in the validation population. Right panel (validation in discovery): Shows the *P*-value distribution of SNPs identified in the validation population when tested in the discovery population


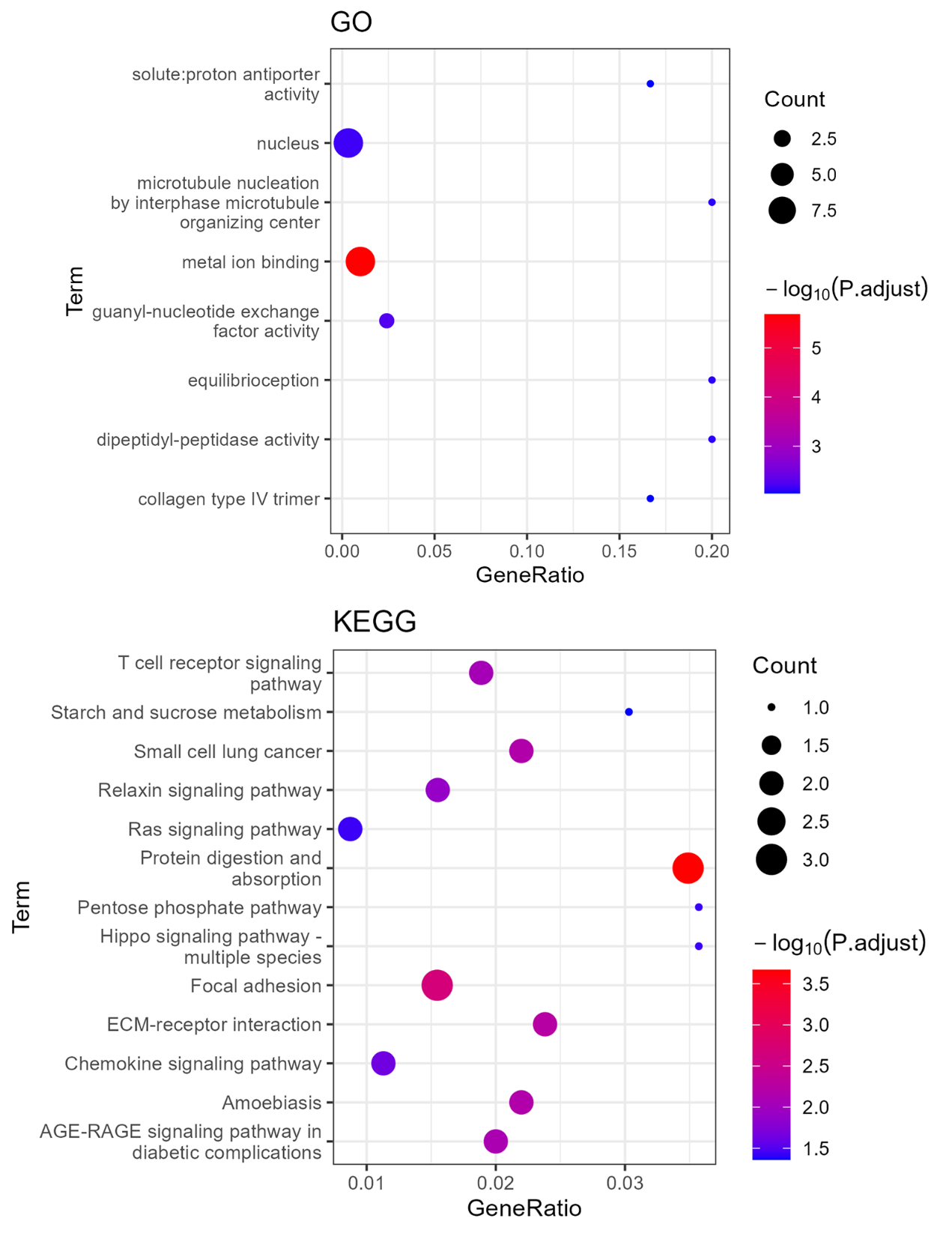


**Fig. S7** The significant enrichment of GO terms and KEGG pathways for individual GWAS in all semen quality traits

**
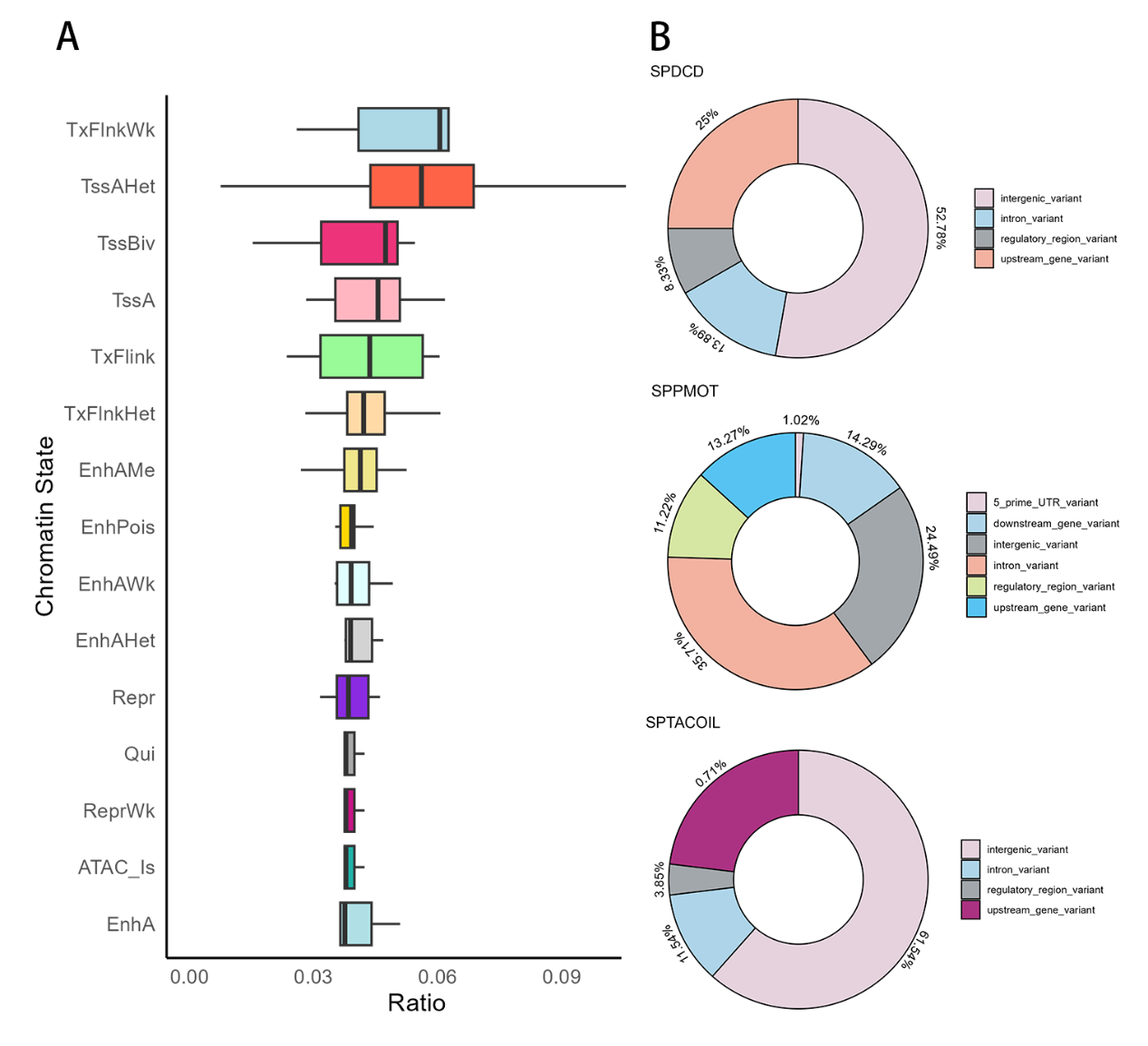
**

**Fig. S8** Functional annotation and variant classification for SPPMOT, SPDCD, and SPTACOIL. **A** The enrichment of SNPs within the QTLs corresponding to the 3 semen quality traits (SPPMOT, SPDCD, and SPTACOIL) from the GWAS in regulatory elements across 14 different tissues. **B** Enrichment of SNPs across different variant types for the SPDCD, SPPMOT, and SPTACOIL


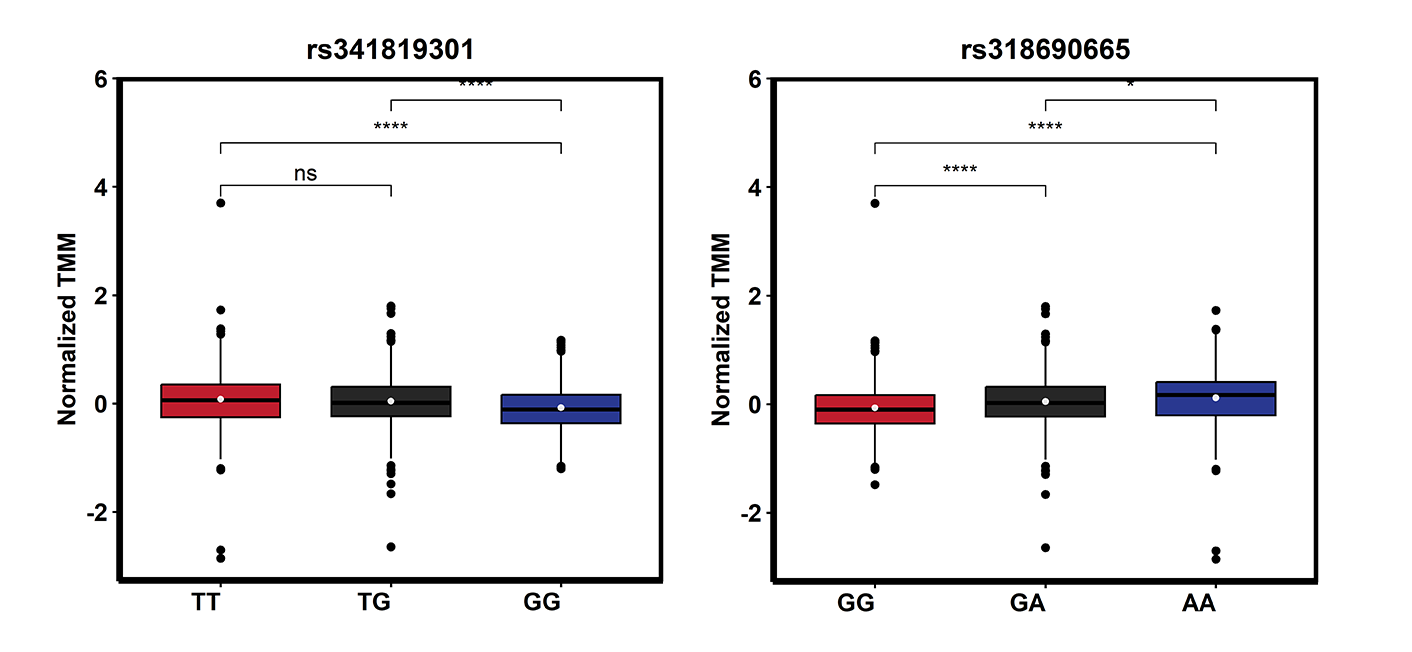


**Fig. S9** The most significant colocalization signal (rs341819301) of *DCAF12* and the top eQTL (rs318690665) of *DCAF12* significantly contribute to the regulation of *DCAF12* expression levels

**
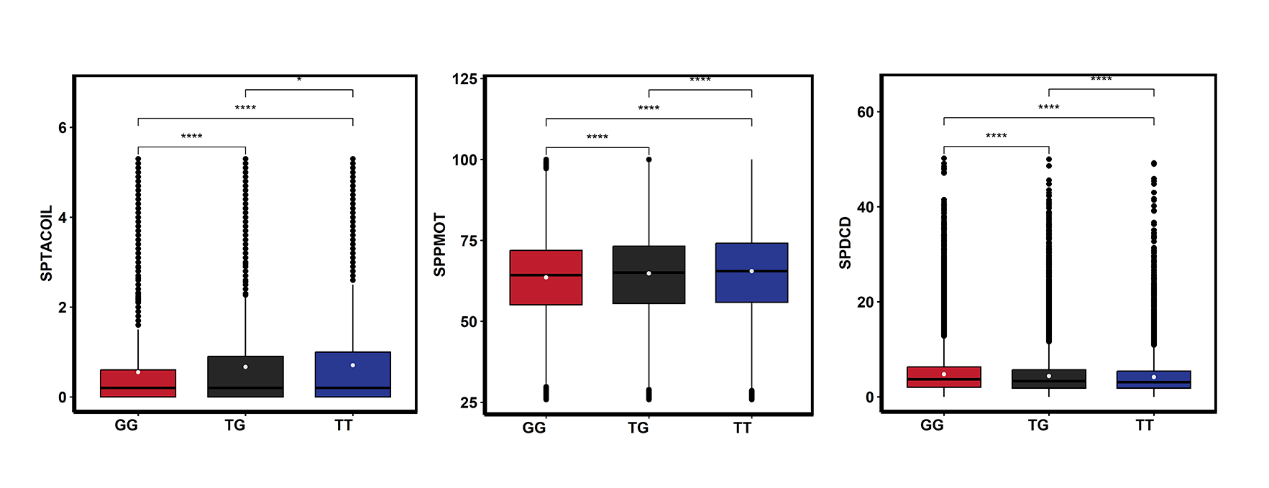
**

**Fig. S10** The effect of the three different genotypes of rs341819301 on the SPTACOIL, SPPMOT, and SPDCD phenotypic variation


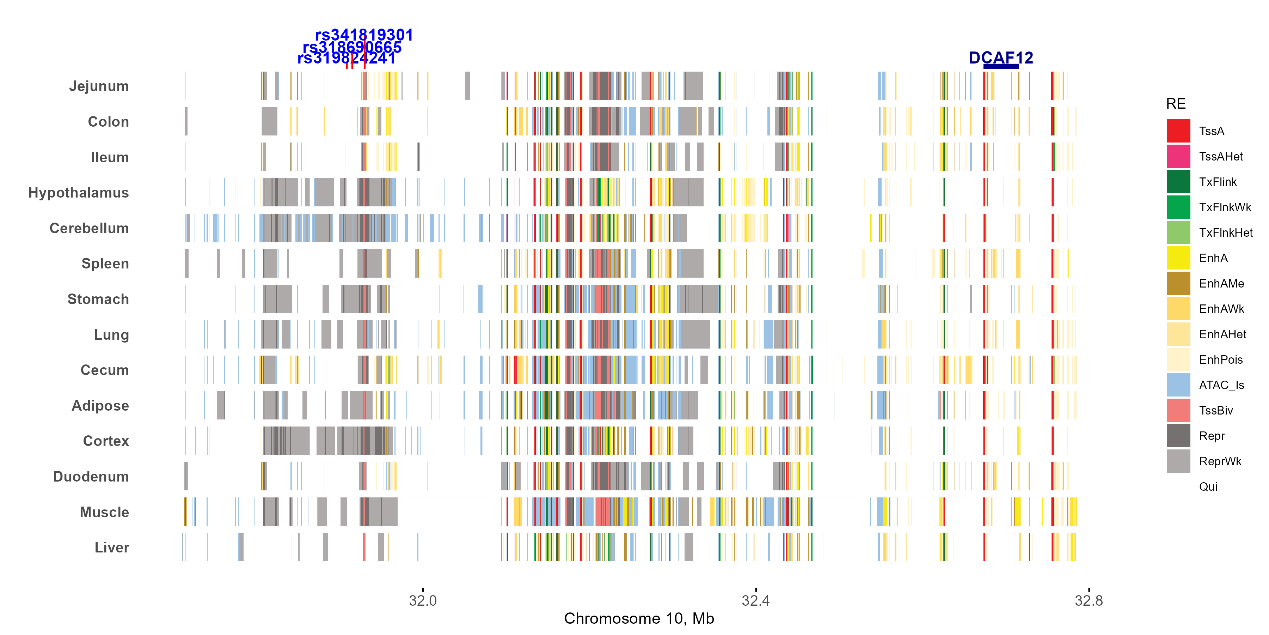


**Fig. S11** The chromatin states of each tissue in the candidate QTL region identified in the GWAS results for SPPMOT, SPTACOIL, and SPDCD are shown. The chromatin states of key regulatory SNPs are indicated by the red rectangle, while the blue rectangle highlights chromatin state elements overlapping with DCAF12, with each color representing distinct regulatory element types

**
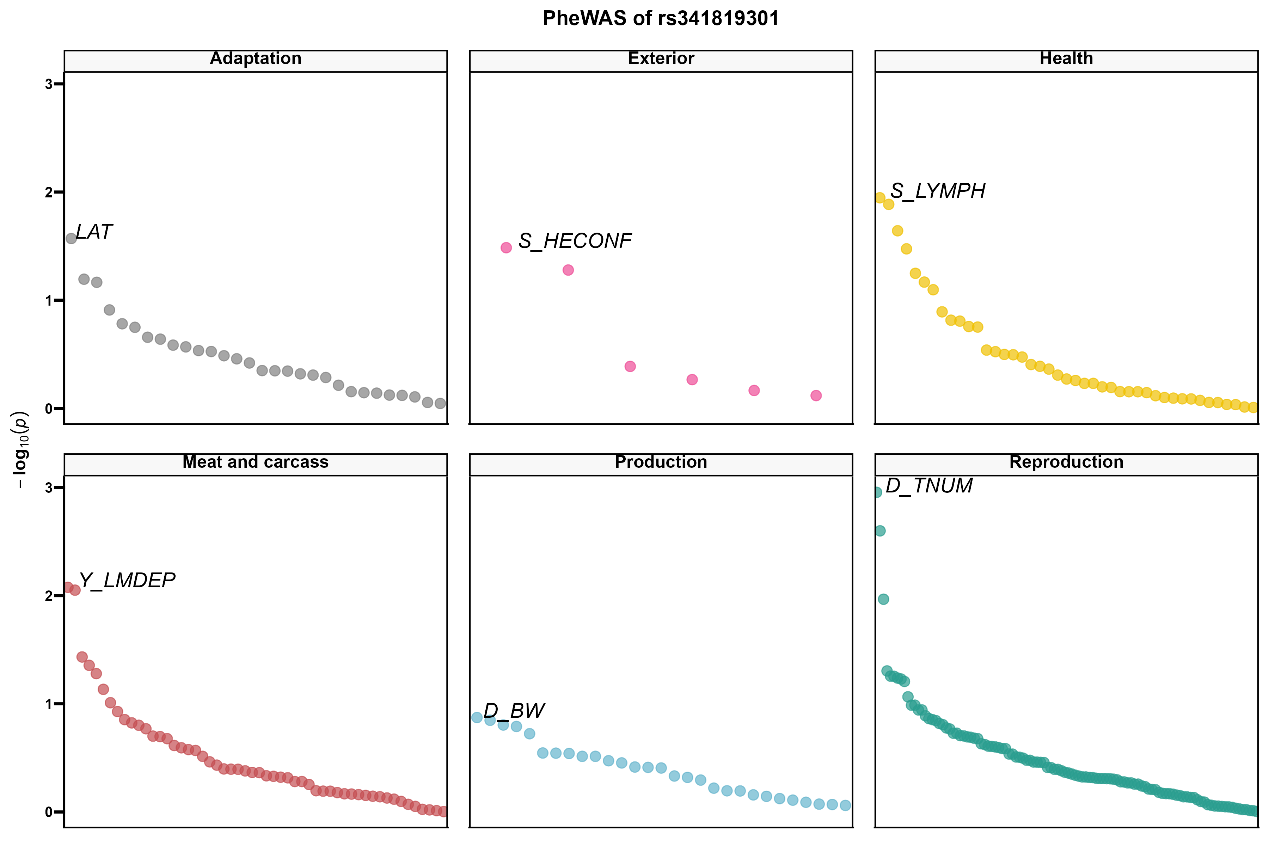
**

**Fig. S12** The PheWAS of rs341819301 in pig. The x-axis represents the different phenotypes within each trait category. The label represents the most significant phenotype for each trait category


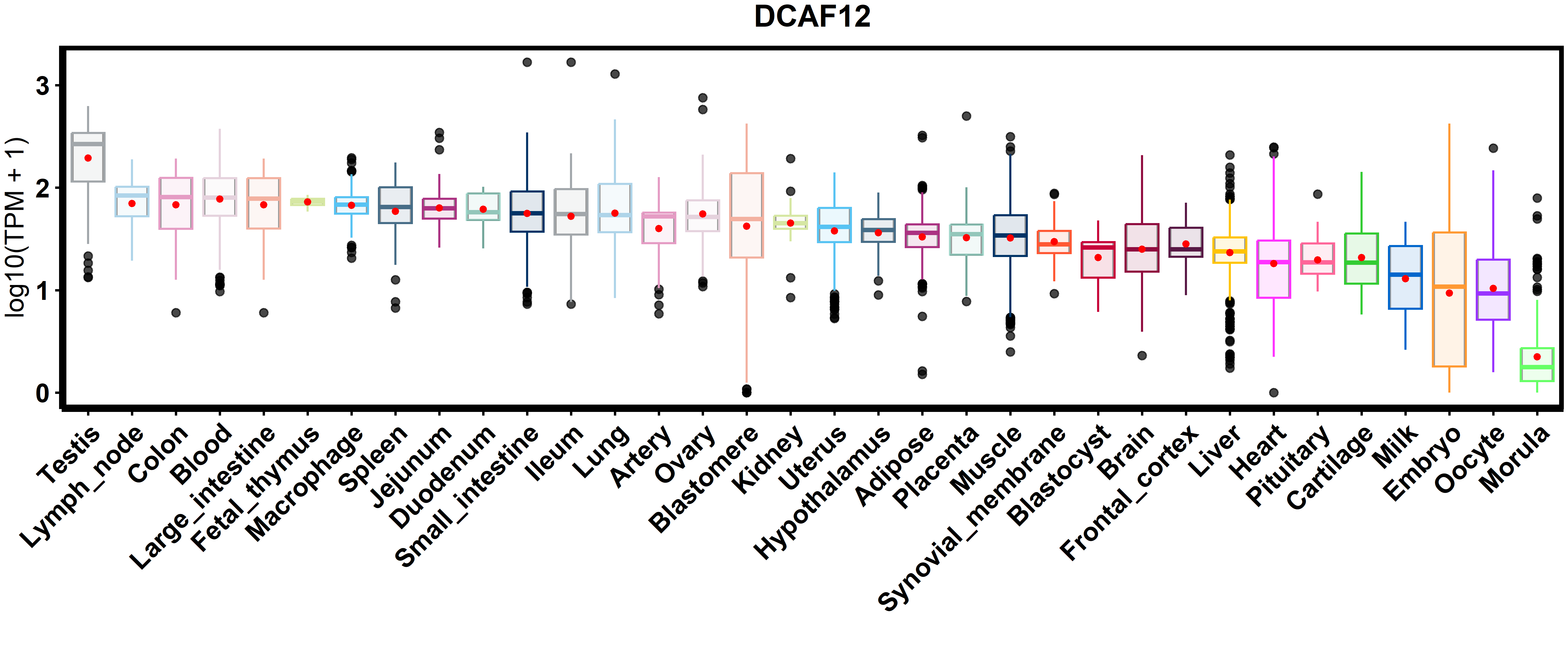


**Fig. S13** The expression level of *DCAF12* in multiple tissues. The x-axis represents tissue types. The y-axis represents the expression level of *DCAF12*


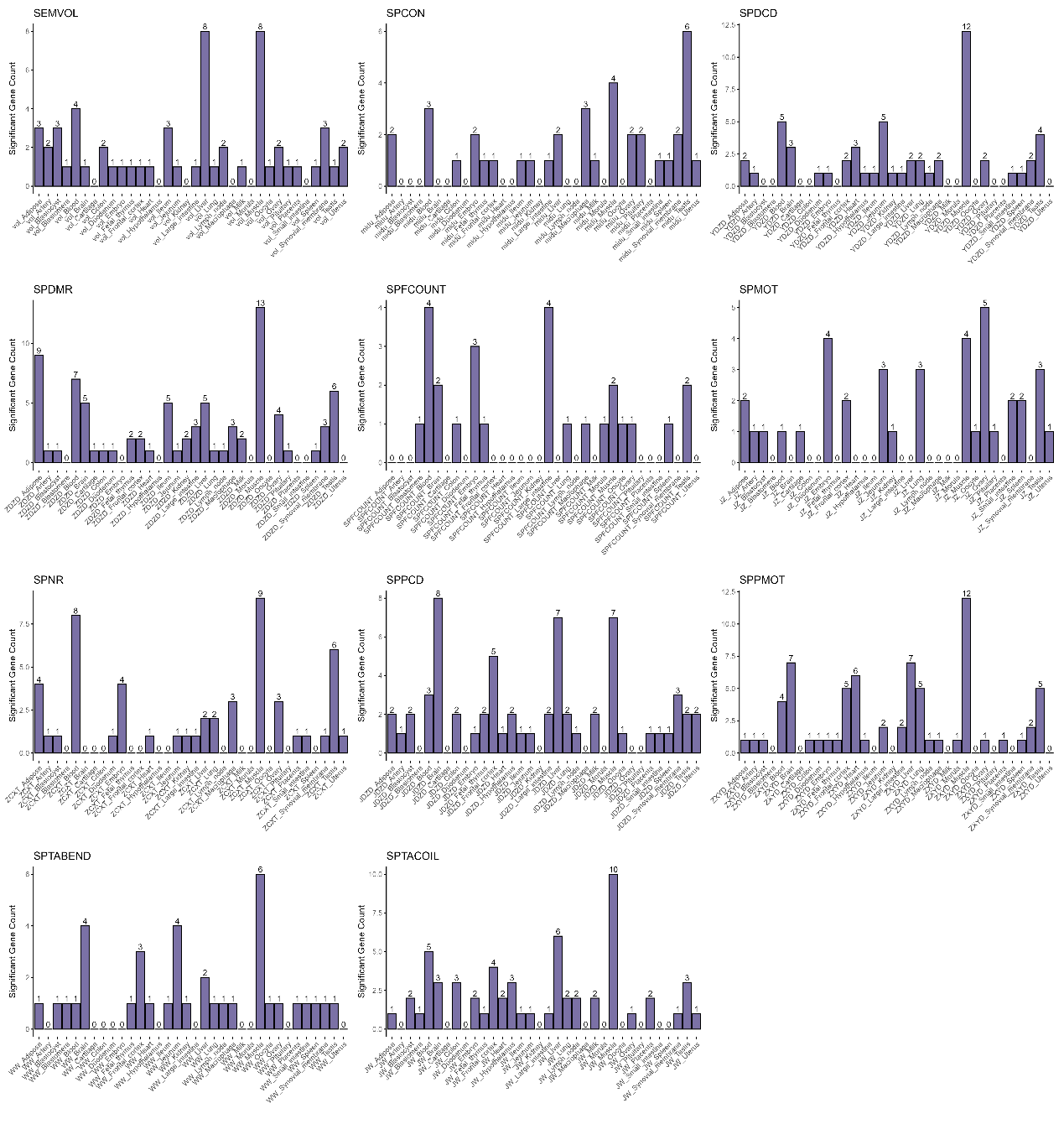


**Fig. S14** The number of significant gene-tissue-phenotype pairs identified in each tissue for TWAS of each trait


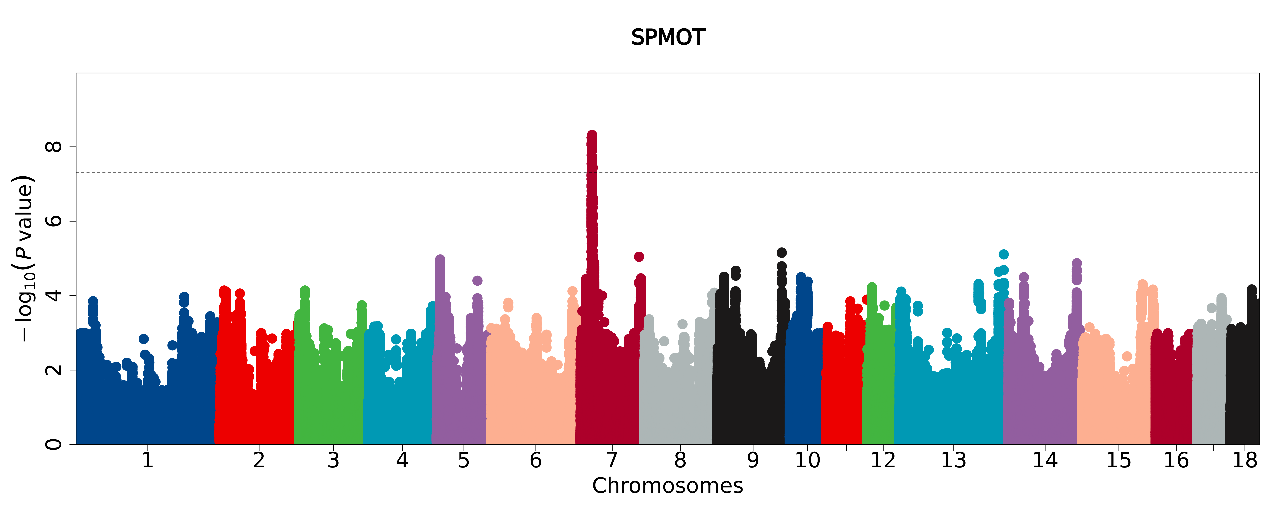


**Fig. S15** The Manhattan plot for SPMOT GWAS. The grey dashed line represents the suggestive significant threshold (*P* = 5.00×10^-8^)


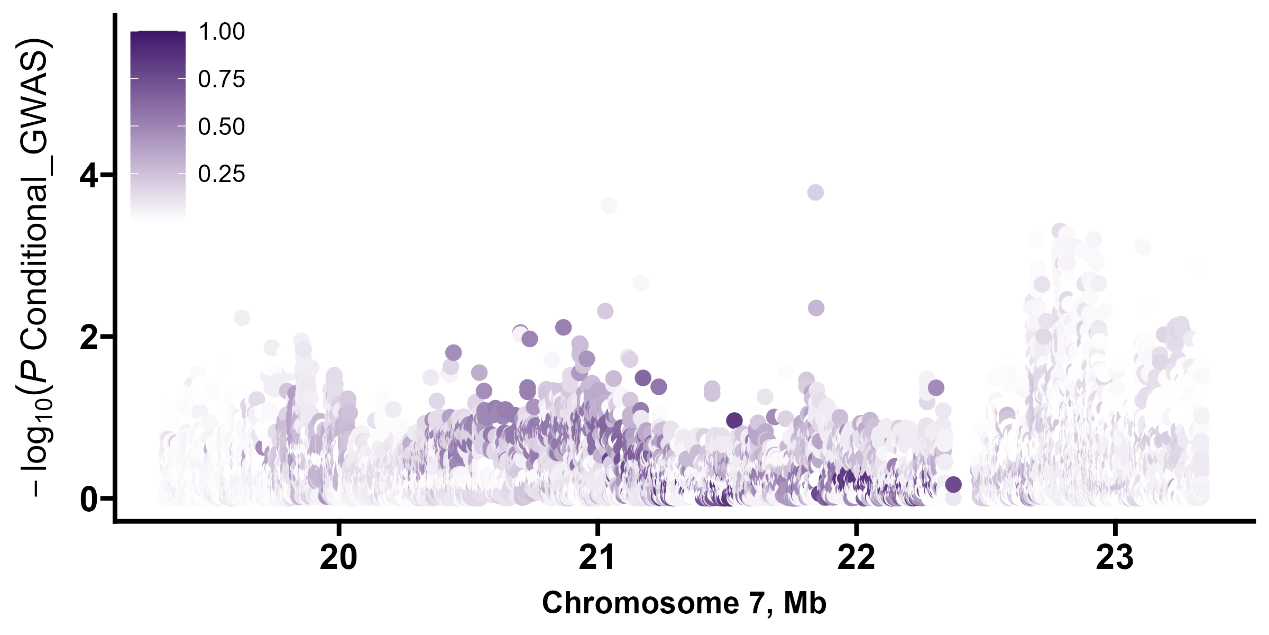


**Fig. S16** The conditional GWAS regional Manhattan plot for rs1112922792 in SPMOT

**
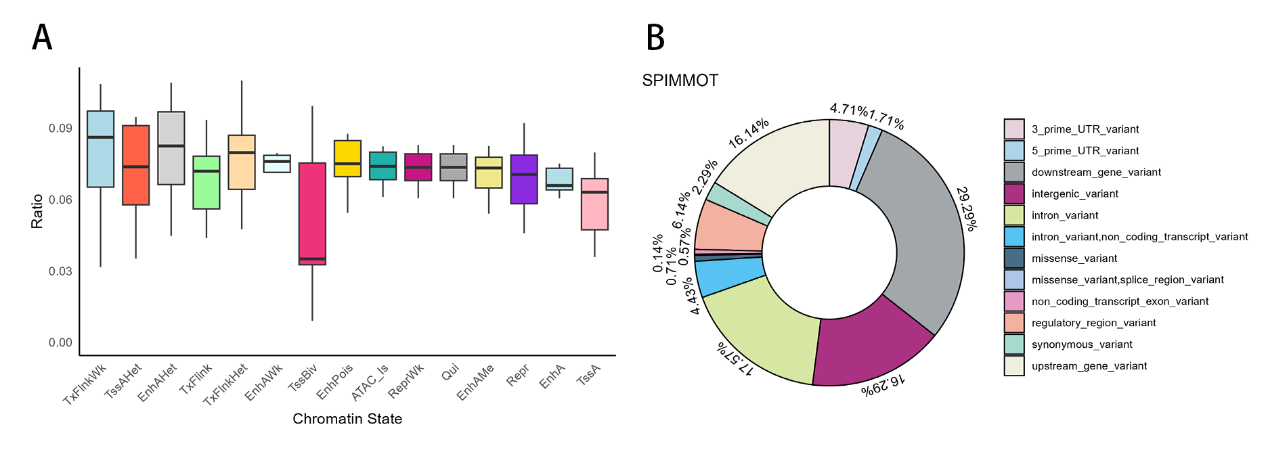
**

**Fig. S17** Functional annotation and variant classification for SPMOT. **A** The SNPs within the QTLs of SPMOT from the GWAS are enriched in regulatory elements of 14 different tissues. **B** Enrichment of SNPs across different variant types for the SPMOT


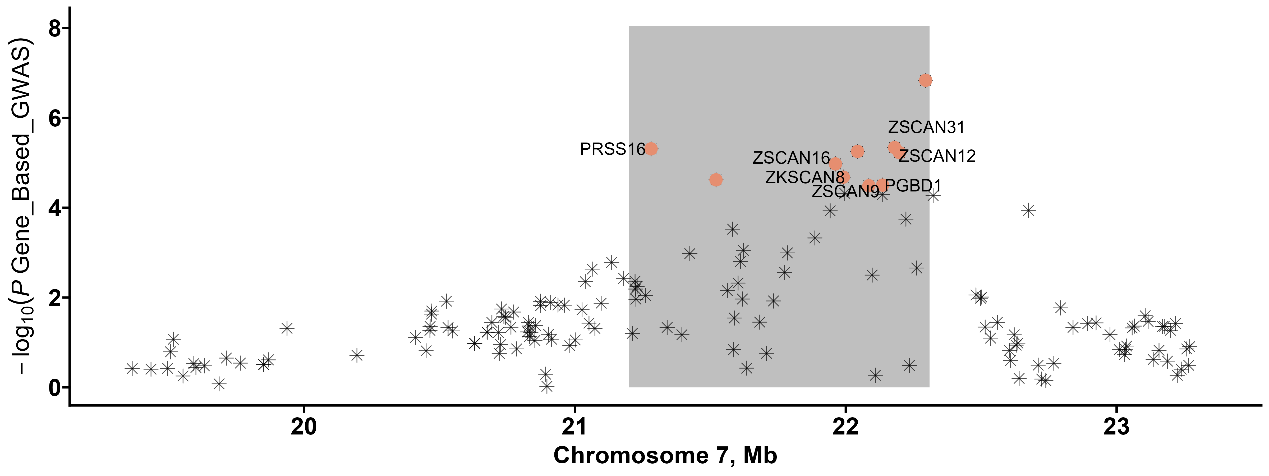


**Fig. S18** The gene-based association analysis for SPMOT. The orange points and labels represent significant genes in gene-based association analysis (*P* < 33.95×10⁻⁵)


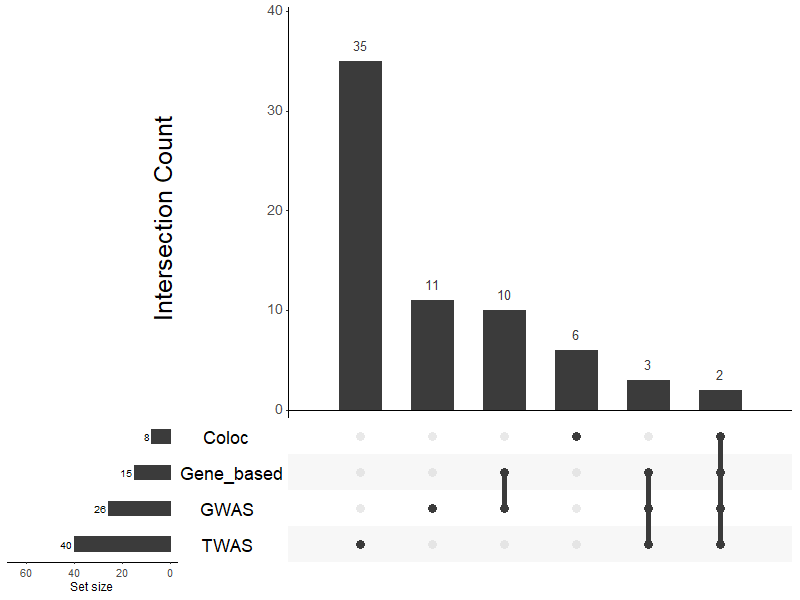


**Fig. S19** The upsetR summary of GWAS and post-GWAS in SPMOT


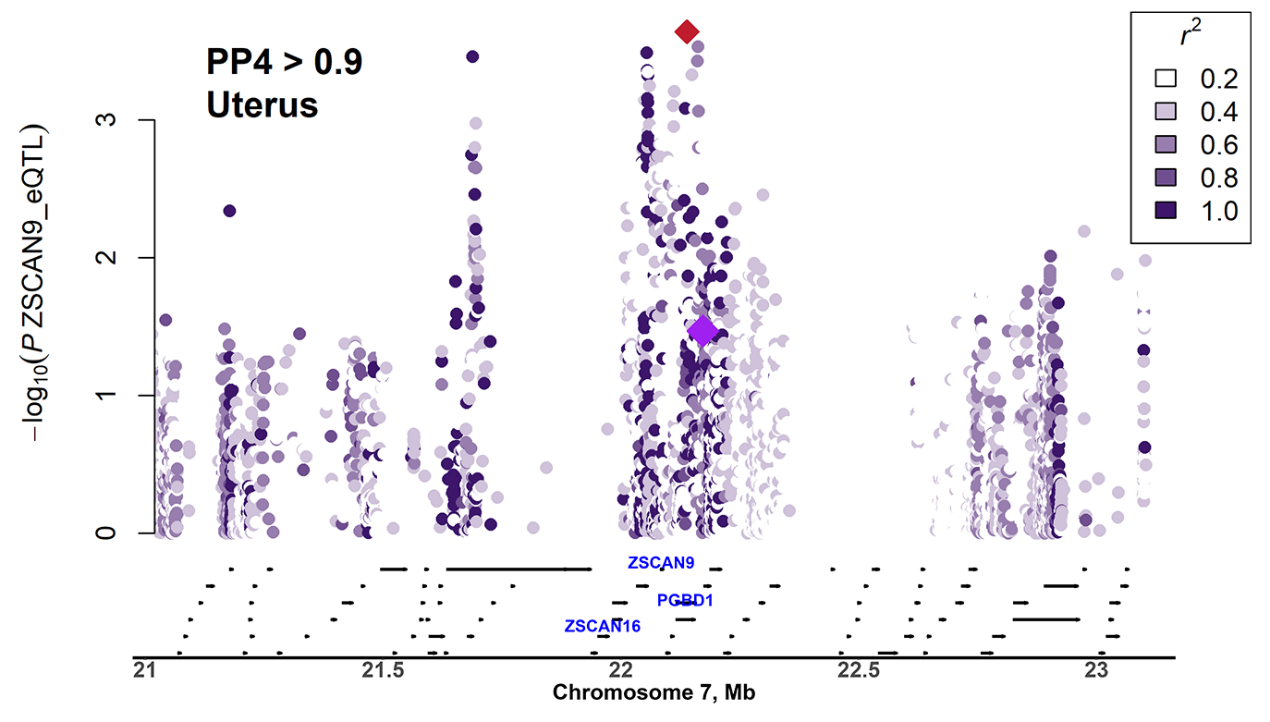


**Fig. S20** The eQTL mapping results for *ZSCAN9* in uterus tissue. The colors correspond to the LD (R2) between independent variant and other variants. The red diamond point represents the lead eQTL of *ZSCAN9* in uterus. The purple diamond point represents the Colocalization loci rs322211455

**
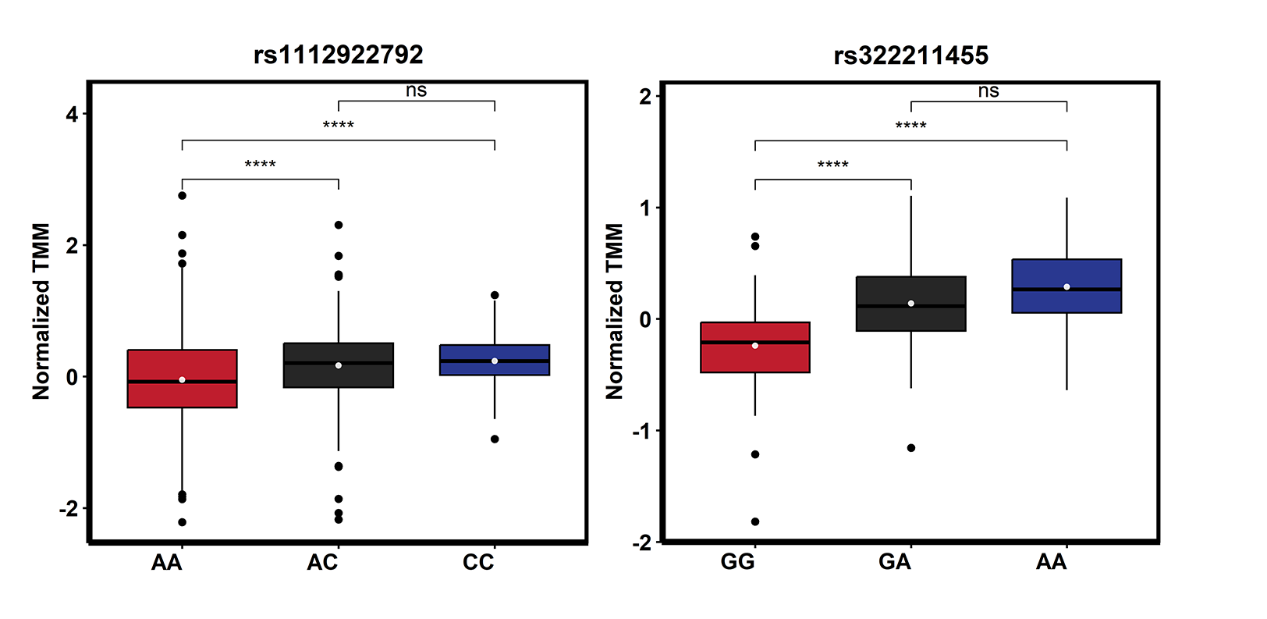
**

**Fig. S21** The most significant colocalization signal (rs322211455) of *ZSCAN9* and top eQTL (rs1112922792) of *ZSCAN9* significantly contribute to the regulation of *ZSCAN9* expression levels

**
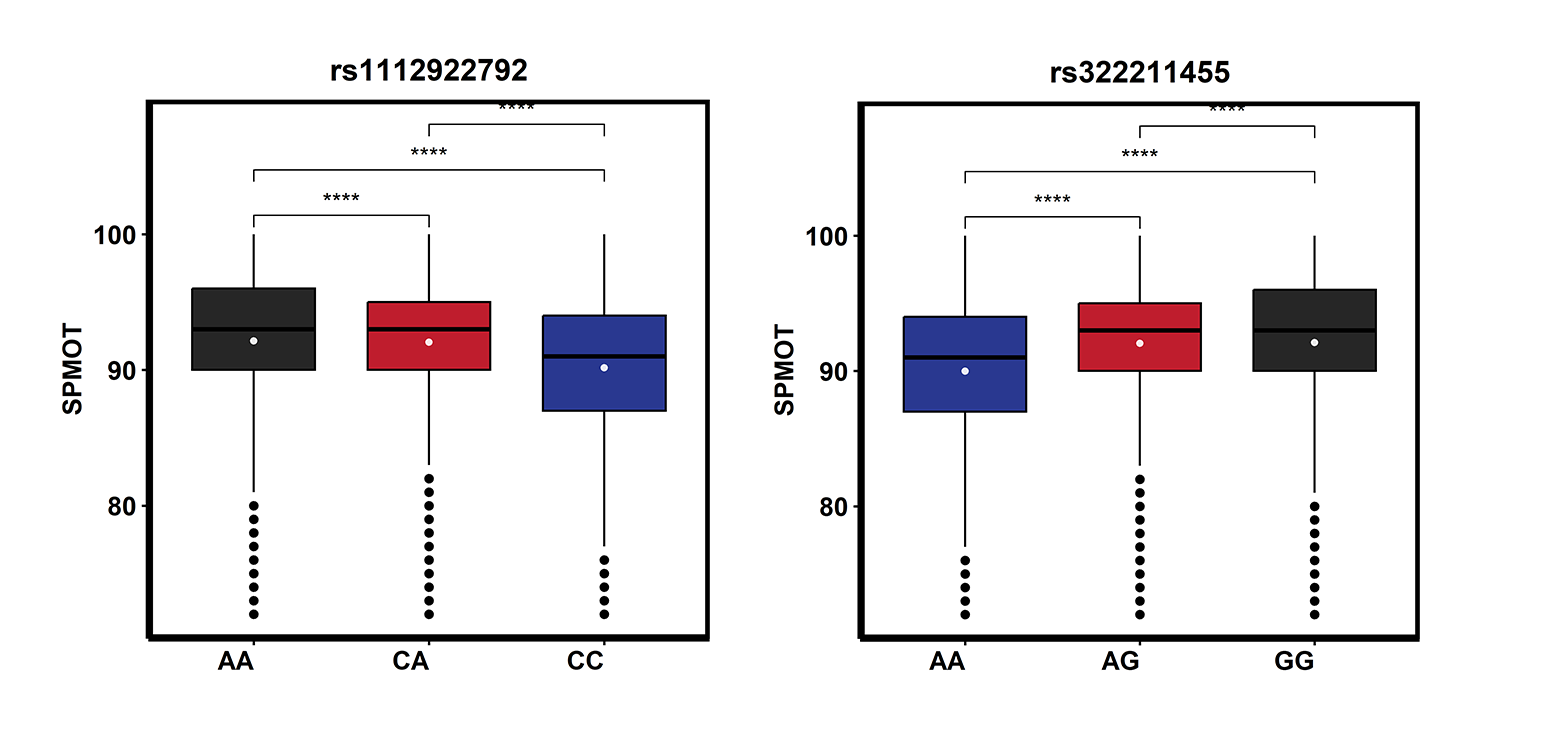
**

**Fig. S22** The impact of the three genotypes of rs322211455 (the colocalization signal in *ZSCAN9*) and rs1112922792 (the lead SNP in the SPMOT GWAS) on the phenotypic variation of SPMOT


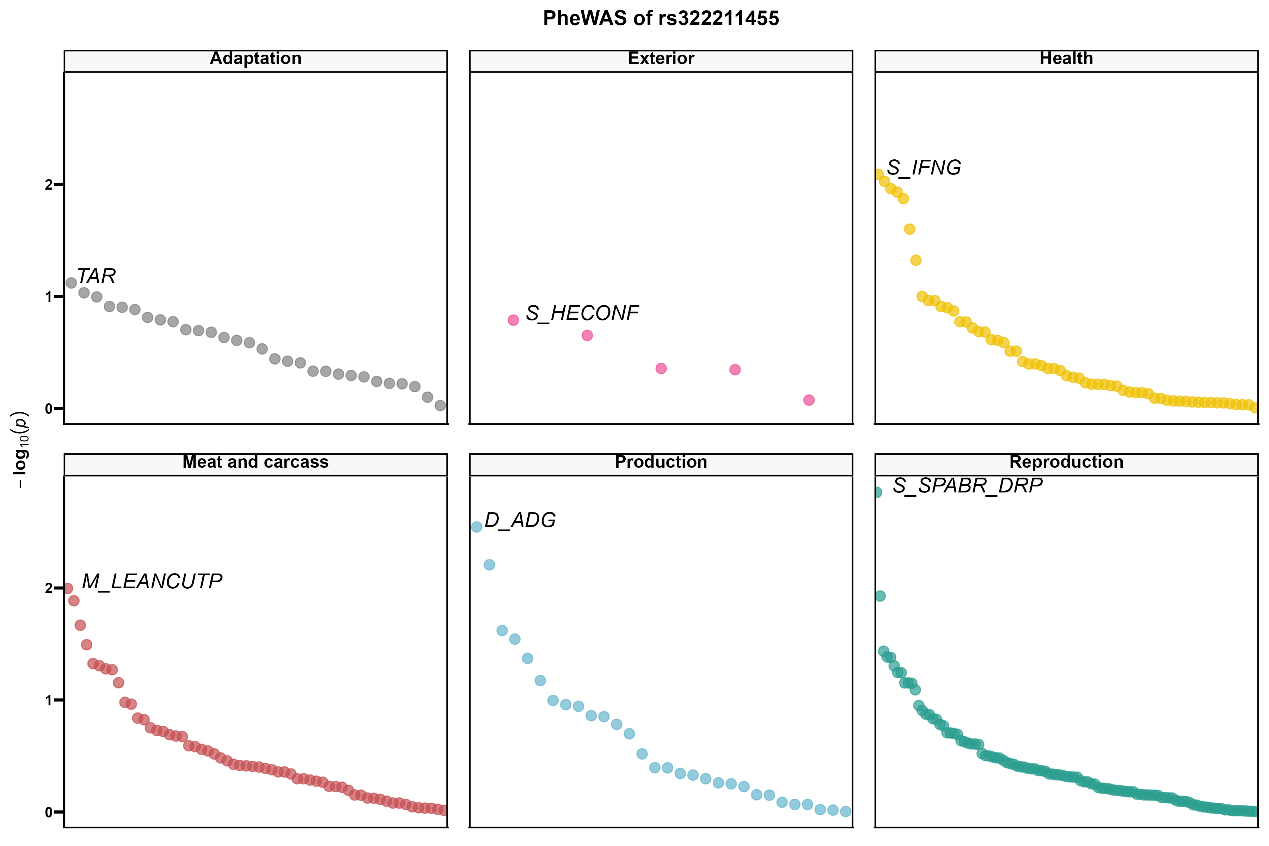


**Fig. S23** The PheWAS of rs322211455 in pig. The x-axis represents the different phenotypes within each trait category. The label represents the significant phenotype for each trait category

**
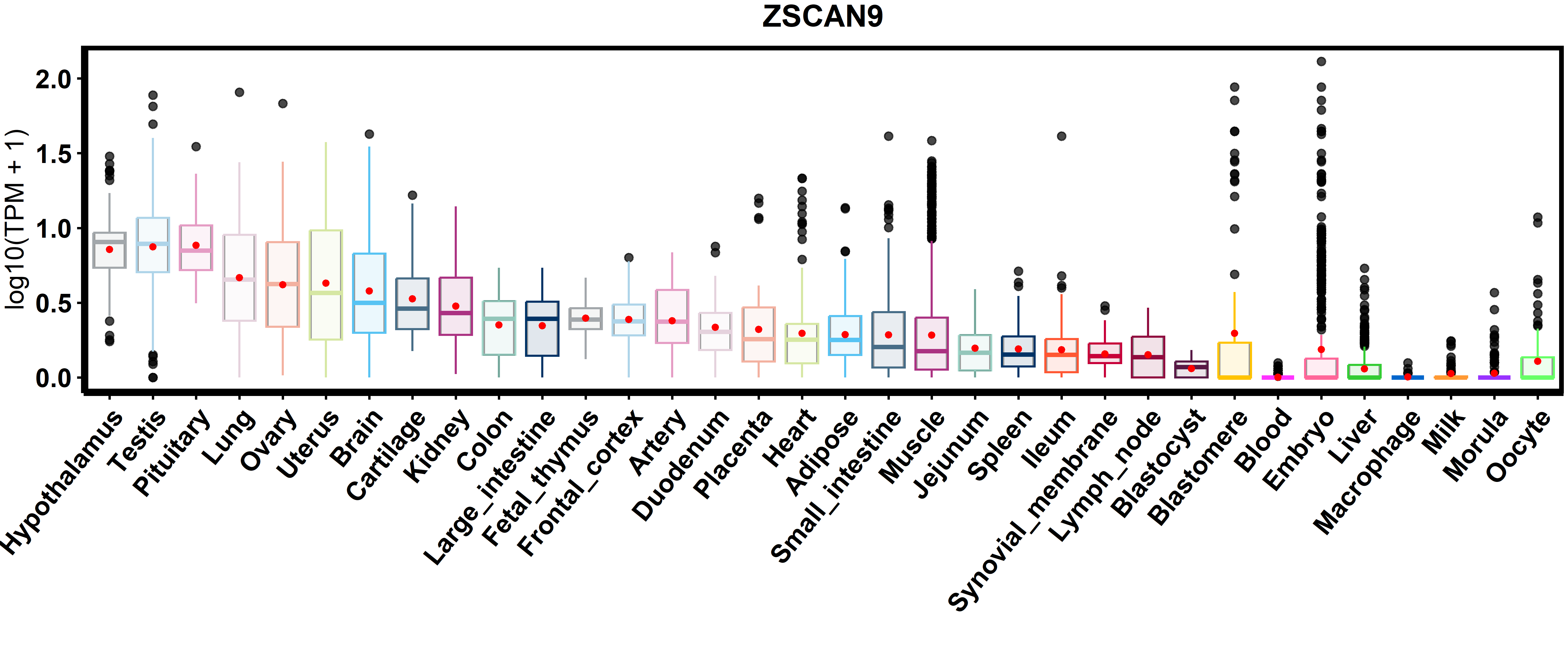
**

**Fig. S24** The expression level of *ZSCAN9* in multiple tissues. The x-axis represents tissue types. The y-axis represents the expression level of *ZSCAN9*
